# Integrated multi-omics profiling reveals immune-related biomarkers and regulatory networks for early prediction of tuberculosis in type 2 diabetes mellitus

**DOI:** 10.3389/fimmu.2026.1755184

**Published:** 2026-02-26

**Authors:** Zhaoyang Ye, Guangliang Bai, Peng Cheng, Cong Peng, Ling Yang, Li Zhuang, Linsheng Li, Yufeng Li, Ruizi Ni, Shuang Zhou, Yajing An, Mingming Zhang, Yuan Tian, Liang Wang, Wenping Gong

**Affiliations:** 1 Senior Department of Tuberculosis, Chinese PLA General Hospital, Beijing, China; 2 Department of Geriatrics, The Eighth Medical Center of PLA General Hospital, Beijing, China; 3 Department of Clinical Laboratory, The Eighth Medical Center of PLA General Hospital, Beijing, China; 4 Handan Municipal Centre for Disease Prevention and Control, Handan, Hebei, China; 5 Graduate School, Hebei North University, Zhangjiakou, Hebei, China

**Keywords:** ceRNA network, early diagnostic model, immune-metabolic dysregulation, multi-omics biomarkers, precision intervention, type 2 diabetes-tuberculosis

## Abstract

**Background:**

Type 2 diabetes mellitus (T2DM) significantly elevates the risk of tuberculosis (TB); however, early detection in T2DM patients is still insufficient. This study aimed to identify immune-based early-warning biomarkers, develop robust prognostic models, and elucidate the immune-metabolic circuitry underlying the comorbidity of type 2 diabetes and tuberculosis (T2DM-TB).

**Methods:**

A prospective cohort study (n = 198; HC 71, T2DM 67, T2DM-TB 60) was conducted, involving whole-transcriptome and plasma-proteome profiling. Differential expression analysis, weighted gene co-expression network analysis (WGCNA), and mining of the ImmPort database facilitated the extraction of immune-relevant genes. Protein-protein interaction (PPI) and competing endogenous RNA (ceRNA) networks were utilized to delineate core regulators. Eleven logistic regression models were developed based on 13 cross-platform biomarkers. The robustness of these models was evaluated through 5-fold cross-validation, and feature selection was optimized using least absolute shrinkage and selection operator (LASSO) regression. External validation was performed using GEO datasets (GSE181143, GSE114192) and reverse transcription quantitative polymerase chain reaction (RT-qPCR). Functional annotation and xCell immune-infiltration analyses were employed to characterize microenvironmental shifts, while dual-luciferase assays confirmed ceRNA interactions.

**Results:**

Thirteen immune-related biomarkers were identified, comprising 4 mRNAs (IRF1, FPR1, LILRB3, SECTM1), 2 microRNAs (miRNAs) (hsa-miR-4726-5p, novel-miR-109), 3 long non-coding RNAs (lncRNAs) (MSTRG.128052.1, MSTRG.4908.1, MSTRG.37670.90), and 4 proteins (IFN-γ, IL-6, CXCL10, CXCL6). Eleven models demonstrated high diagnostic efficacy, with area under the curve (AUC) values ranging from 0.93 to 0.99, and exhibited stable performance in 5-fold cross-validation, yielding AUC values between 0.77 and 0.95. LASSO-derived concise biomarker subsets overlapped with primary model features, thereby confirming robust discriminative stability. Gene ontology (GO) and Kyoto Encyclopedia of Genes and Genomes (KEGG) analyses underscored the significance of immune response, inflammation, and metabolic regulation, highlighting key pathways such as Toll-like receptors, NF-κB, and JAK-STAT. Immune infiltration analysis revealed a “pro-inflammatory-suppressive-reconstructive” imbalance characterized by overactivated innate immunity, including M1/M2 macrophages and NKT cells, alongside compromised adaptive immunity, evidenced by reduced CD4⁺/CD8⁺ T cells and B cells. Additionally, ceRNA networks and dual-luciferase assays confirmed that novel-miR-109 inhibits the translation of FPR1, LILRB3, and MSTRG.4908.1, while hsa-miR-4726-5p targets the 3’ UTR of SECTM1.

**Conclusions:**

This study establishes a validated multi-omics framework for the early detection of T2DM-TB, elucidates key regulatory axes (IRF1/IFN-γ, ceRNA circuitry, CXCL10/CXCL6), and provides actionable biomarkers and high-performance models for precision intervention in T2DM-TB management.

## 1 Introduction

Diabetes mellitus (DM) is a metabolic disorder marked by chronic hyperglycemia, which presents a substantial global health challenge. Type 2 diabetes mellitus (T2DM) constitutes over 90% of all diabetes cases worldwide (1). The International Diabetes Federation reported that in 2021, approximately 537 million adults aged 20–79 were living with diabetes, resulting in a prevalence of 10.5% and 6.7 million deaths; notably, 32.6% of these deaths occurred in individuals under 60 years old (2). A subsequent study published in The Lancet in 2022 estimated the global number of adult patients to be 828 million (3). In addition to causing multi-system complications, T2DM significantly impairs immune function, making individuals more vulnerable to infections, including *Mycobacterium tuberculosis* (MTB) (4, 5).

Tuberculosis (TB), resulting from MTB infection, continues to be the foremost cause of death attributable to a single infectious agent (6–9). According to the World Health Organization’s 2025 report, there were 10.7 million new TB cases and 1.23 million deaths worldwide in 2024 (10). China represents 6.5% of global cases, following India (25%), Indonesia (10%), and the Philippines (6.8%) (10).

Substantial evidence identifies T2DM as a significant independent risk factor for TB (4–6, 11–13). Patients with T2DM exhibit more than a three-fold increased risk of developing active TB compared to the general population (14). Furthermore, the comorbidity of TB and diabetes (T2DM-TB) is associated with more severe disease manifestations, including higher bacterial loads, extensive radiological involvement (e.g., cavitation, infiltration, fibrosis, or multifocal lesions), and prolonged treatment duration (15–23). T2DM also markedly elevates the risk of drug-resistant TB (DR-TB). A meta-analysis involving 15 countries confirmed a significant association between T2DM and DR-TB (24), with some studies reporting a 2 to 8-fold increased risk of DR-TB among patients with T2DM-TB (17, 25–29). Additionally, T2DM may disrupt the metabolism of anti-tuberculosis drugs, diminishing treatment efficacy and resulting in significantly higher rates of treatment failure and relapse (5, 30). Compared to patients with TB alone, those with T2DM-TB face a four-fold increased risk of relapse (31–33) and a two-fold increased risk of mortality (34). Modeling studies indicate that sustained reductions in T2DM incidence prior to 2025 could prevent 7.8 million new TB cases and 1.5 million TB-related deaths by 2035 (35). However, systematic screening approaches for TB risk among individuals with diabetes are still lacking, leading to substantial delays in recognizing and addressing high-risk cases. Consequently, there is a critical necessity to exploit multi-omics methodologies and high-throughput screening to pinpoint early predictive biomarkers for the T2DM-TB. These biomarkers can then be used to develop risk assessment models that facilitate accurate identification and tailored management of individuals at high risk of TB within the T2DM cohort.

In recent years, rapid advancements in high-throughput omics technologies have equipped researchers with powerful tools for elucidating disease mechanisms and identifying biomarkers (9, 36). Transcriptomics, through comprehensive profiling of gene expression, can uncover critical molecular events involved in disease pathogenesis and progression. Biomarkers identified via transcriptomic screening, such as ESR1, PGR, and ERBB2, are already extensively utilized in molecular subtyping and treatment decision-making for breast cancer (37). Proteomics, which directly measures protein expression, modifications, and functional states, provides a more accurate representation of the physiological changes occurring in cells under pathological conditions (38, 39).

This study integrates whole-transcriptome and plasma proteome data with bioinformatics approaches to identify and elucidate potential immune-related biomarkers for T2DM-TB. We utilized Weighted Gene Co-expression Network Analysis (WGCNA) to identify key gene modules, cross-referenced these with immune-related genes from the ImmPort database, and employed Protein-Protein Interaction (PPI) networks to identify core regulatory proteins. An early-risk prediction model was constructed using logistic regression, and the biomarkers were validated through GEO dataset mining and RT-qPCR experiments. Functional annotations through Gene Ontology (GO) and Kyoto Encyclopedia of Genes and Genomes (KEGG) enrichment analyses clarified the biological roles of these molecules, while lymphocyte subset enumeration and immune infiltration analysis characterized the immune microenvironment. Additionally, we established a competing endogenous RNA (ceRNA) regulatory network and validated key interactions using dual-luciferase reporter assays. Our work enhances the understanding of T2DM-TB mechanisms, providing a theoretical and practical foundation for precision diagnosis and treatment, while also revealing characteristics of the patient immune microenvironment that suggest potential targets for immune intervention. Future research may integrate single-cell sequencing, multi-omics data, and large-scale clinical validation to refine risk prediction models, thereby offering scientific guidance for TB prevention and control in patients with T2DM.

## 2 Materials and methods

### 2.1 Study cohort, ethics, and clinical characterization

This prospective, single-center cohort study was conducted at the Eighth Medical Center of the Chinese PLA General Hospital from October 2022 to August 2024. Participants were divided into three groups: healthy controls (HCs), patients with T2DM, and patients with T2DM-TB. All participants provided written informed consent before enrollment. The study protocol received approval from the Institutional Review Board of the Eighth Medical Center of the PLA General Hospital (Approval No: 3092023122013297234) and was conducted in accordance with the principles outlined in the Declaration of Helsinki.

The detailed inclusion and exclusion criteria were as follows:

1. HCs: Individuals aged 18–60 years with no history of TB exposure or clinical symptoms, normal chest imaging, a negative interferon-γ release assay, and a negative HIV test. Individuals with a prior history of TB, long-term residence in high-risk areas, HIV positivity, active malignancy, or autoimmune diseases were excluded.
2. Patients with T2DM: Diagnosis was established according to the 2020 Chinese Diabetes Society guidelines (40). Individuals aged 18–60 years were included. Those with HIV, malignancy, autoimmune diseases, severe organ dysfunction, history of immunomodulatory or corticosteroid therapy within the preceding 6 months, or non-T2DM forms of diabetes were excluded.
3. Patients with T2DM-TB: Patients met the diagnostic criteria for both T2DM (as previously described) and pulmonary tuberculosis according to the WS 288–2017 Chinese national standard (41), aged 18–60 years. The exclusion criteria were identical to those of the T2DM group.

A total of 624 individuals were screened, resulting in the enrollment of 198 subjects who met the specified criteria. Basic clinical characteristics, such as age and sex, were collected for all participants.

### 2.2 Sample collection, processing, and omics data generation

#### 2.2.1 Peripheral blood lymphocyte subset enumeration

Fifty microliters of whole blood collected in EDTA tubes were stained with 10 μL of Multitest™ 6-color TBNK reagent (BD Biosciences) in Trucount™ absolute counting tubes. Following a 15-minute incubation at room temperature in the dark, 450 μL of BD Pharm Lyse™ lysing solution was added, and another 15-minute incubation ensued. Subsequently, the samples were promptly analyzed on a FACSAria II flow cytometer (BD Biosciences) to determine the absolute counts and percentages of T, B, and NK cell subsets. A sequential gating strategy was implemented: lymphocytes were gated based on FSC-A/SSC-A, singlets on FSC-H/FSC-A, and subsets were characterized as T (CD3^+^), helper T (CD3^+^CD4^+^), cytotoxic T (CD3^+^CD8^+^), B (CD3^−^CD19^+^), and NK (CD3^−^CD16/56^+^) cells. Absolute counts were calculated utilizing Trucount™ beads.

#### 2.2.2 Peripheral blood mononuclear cell isolation and RNA extraction

Peripheral blood was diluted 1:1 with PBS and carefully layered over Ficoll-Paque™ PLUS (Cytiva). Centrifugation was conducted at 2,500 rpm for 20 minutes at room temperature. The layer of PBMCs was collected, washed twice with RPMI-1640 medium (1,500 rpm for 10 minutes), and resuspended in AIM-V serum-free medium (Gibco). The cell count was adjusted to 2.5×10⁶ cells/mL, and aliquots were stored at –80 °C until needed. Total RNA was extracted from the PBMCs using Trizol reagent. RNA concentration was quantified using a NanoDrop 2000 (Thermo Fisher Scientific), and integrity was evaluated with an Agilent 2100 Bioanalyzer. Only samples with an RNA Integrity Number (RIN) ≥ 7.0 were utilized for subsequent transcriptome sequencing.

#### 2.2.3 Whole-transcriptome sequencing

Ribosomal RNA was depleted utilizing the NEBNext^®^ rRNA Depletion Kit. Libraries for lncRNA and mRNA were constructed in accordance with the manufacturer’s protocol, which included fragmentation, double-stranded cDNA synthesis, end-repair/A-tailing, adapter ligation, and PCR amplification. Small RNA (miRNA) libraries were prepared using the Nextflex™ Small RNA Kit, which encompassed 3’ and 5’ adapter ligation, reverse transcription, and PCR amplification. Fragments ranging from 140 to 160 bp were size-selected and purified through 6% PAGE gel electrophoresis. All libraries underwent quality control using a Qsep-400 system and were sequenced on an Illumina NovaSeq 6000 platform employing a 2×150 bp paired-end strategy.

#### 2.2.4 Targeted proteomics

Plasma samples were subjected to centrifugation at 3,000 rpm for 5 minutes, after which the supernatants were analyzed using the Olink Target 96-plex panel (Olink Proteomics, Olink Target 96 Inflammation) in accordance with the standard Proximity Extension Assay (PEA) protocol. Data acquisition was performed on a Fluidigm Biomark

HD system. Protein abundances were expressed as Normalized Protein eXpression (NPX) values, a relative log2-scale unit specific to Olink.

### 2.3 Bioinformatics and statistical analysis

#### 2.3.1 Data preprocessing and differential expression analysis

Raw RNA-seq reads underwent quality control and adapter trimming using Trimmomatic. Clean reads were aligned to the GRCh38 human reference genome with HISAT2, and transcript abundance was quantified using StringTie. Novel miRNAs were predicted from small RNA-seq data via miRDeep2. Gene and miRNA expression levels were normalized to Transcripts Per Million (TPM). Differential expression analysis for mRNAs, lncRNAs, miRNAs, and proteins was conducted using the DESeq2 (v1.38.3) package in R. Features exhibiting an absolute fold change ≥ 1.5 and an adjusted p-value (Benjamini-Hochberg False Discovery Rate, FDR) < 0.05 were deemed significantly differentially expressed. Volcano plots were generated using ggplot2 in R. Additionally, to integrate TPM-based RNA expression and log2 NPX-based protein expression into the same logistic regression models, z-score standardization was applied to all molecular features. RNA features were first log2-transformed (log2(TPM + 1)) prior to standardization, while protein features were standardized directly using log2 NPX values to eliminate scale differences while preserving relative expression patterns.

#### 2.3.2 Functional enrichment and network analysis

Enrichment analyses for differentially expressed genes (DEGs) were performed using the ClusterProfiler R package, with terms having an adjusted p-value < 0.05 considered significantly enriched. Subsequently, a weighted gene co-expression network was established utilizing the WGCNA R package, selecting a soft-thresholding power (β) of 18 to achieve a scale-free topology fit index (R²) > 0.88. Modules were distinguished through dynamic tree cutting (minimum module size = 30) and merged if their eigengene similarity exceeded 0.75. Module-trait relationships were assessed to pinpoint modules most relevant to the T2DM-TB phenotype. Genes from pivotal modules (module membership > 0.8 and gene significance > 0.1) were cross-referenced with the ImmPort database’s roster of immune-related genes to pinpoint immune-specific hub genes. The Protein-Protein Interaction (PPI) network for differentially expressed proteins (DEPs) was constructed using STRING (v11.5, confidence score ≥ 0.4) and depicted in Cytoscape (v3.9.1). Hub proteins were singled out employing the CytoHubba plugin with the Degree algorithm. Functional associations among hub proteins were further scrutinized using GeneMANIA. The ceRNA network was formulated by foreseeing miRNA-mRNA and miRNA-lncRNA interactions through miRanda and TargetScan. Only interactions involving differentially expressed transcripts were preserved. Significant ceRNA pairs were sieved based on a noteworthy negative correlation and shared miRNA targeting. The ultimate lncRNA-miRNA-mRNA network was visualized in Cytoscape.

#### 2.3.3 Immune cell infiltration and validation

The abundance of 64 immune and stromal cell types was estimated from transcriptome data using the xCell algorithm. Differences in cell type abundances between groups were evaluated using the Wilcoxon rank-sum test or the Kruskal-Wallis test, as appropriate. Spearman correlation analysis was employed to examine cell-cell relationships and associations between biomarker expression and immune cell abundance. For external validation, GEO datasets (GSE181143 and GSE114192) (**Supplementary Table S1**) that met our predefined inclusion criteria—human mRNA datasets containing HC, T2DM, and T2DM-TB groups with n ≥ 3 per group—were selected. The expression levels of the identified mRNA biomarkers were extracted and analyzed. For multi-omics integrative analyses, such as biomarker screening and ceRNA network construction, only subjects from the same cohort with matched whole-transcriptome and plasma proteomics data were included to minimize heterogeneity and ensure cross-platform consistency.

### 2.4 Experimental validation

#### 2.4.1 RT-qPCR validation

RNA and miRNA were reverse transcribed utilizing the FastKing gDNA Dispelling RT SuperMix and the miRcute Plus miRNA First-Strand cDNA Kit (TIANGEN), respectively. Quantitative PCR was conducted on an ABI QuantStudio 5 system. The reaction conditions were as follows: for mRNA—95 °C for 2 min; 40 cycles of 95 °C for 5 s and 60 °C for 25 s; for miRNA—95 °C for 15 min; 40 cycles of 95 °C for 20 s and 60 °C for 34 s; for lncRNA—95 °C for 5 min; 40 cycles of 95 °C for 10 s and 60 °C for 25 s. Melting curve analysis was carried out for all reactions. GAPDH and U6 snRNA were utilized as internal controls for mRNA/lncRNA and miRNA, respectively. Relative expression levels were determined using the 2^–ΔΔCt method. Primer sequences can be found in **Supplementary Table S2**.

#### 2.4.2 Dual-luciferase reporter assay

DNA fragments containing either the wild-type or mutant predicted binding sites for novel-miR-109 (located in FPR1, LILRB3, MSTRG.4908.1) and hsa-miR-4726-5p (found in the SECTM1 3’ UTR) were synthesized and subsequently cloned into the pmirGLO dual-luciferase vector (Promega) utilizing SacI and SbfI restriction sites (**Supplementary Figure S1**). All constructs underwent verification through Sanger sequencing. HEK-293T cells were cultured in 24-well plates and transfected when they reached 50–70% confluency. After 6 hours, the medium was replaced. Firefly and Renilla luciferase activities were assessed 24–48 hours post-transfection using the Dual-Luciferase^®^ Reporter Assay System (Promega). Firefly luciferase activity was normalized to Renilla luciferase activity for each sample.

### 2.5 Model construction and data availability

#### 2.5.1 Logistic regression models

Eleven binary logistic regression models were constructed utilizing z-score standardized multi-omics features, including mRNAs, lncRNAs, miRNAs, and proteins, as independent variables to predict the risk of T2DM-TB. The performance of the models was evaluated through Receiver Operating Characteristic (ROC) curves, and the Area Under the Curve (AUC) was reported along with 95% confidence intervals. Calibration plots were employed to examine the concordance between predicted probabilities and observed outcomes. To enhance stability and reduce the risk of overfitting, five-fold cross-validation was conducted.

#### 2.5.2 LASSO regression

To enhance model robustness and identify a parsimonious feature set, we performed Least Absolute Shrinkage and Selection Operator (LASSO) regression with ten-fold cross-validation on the transcriptomic and proteomic biomarkers separately following our previous studies (8, 42).

#### 2.5.3 Data availability

All other data supporting the findings of this study are available within the article and its **Supplementary Information Files**.

## 3 Results

### 3.1 Cohort characteristics and peripheral immune profiling

**Figure 1:**
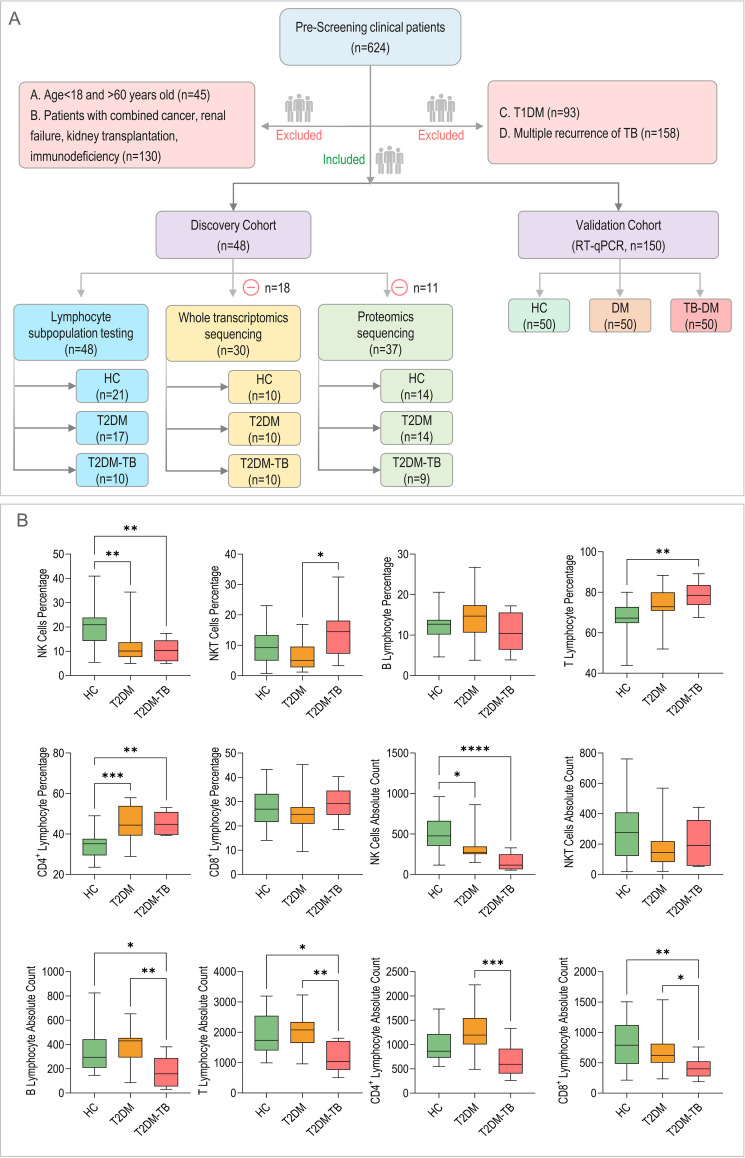
Study overview and peripheral immune profiles. **(A)** The CONSORT flow diagram depicting participant screening, exclusion criteria, and final cohort allocation. Note: Whole-transcriptome sequencing and plasma proteomics were performed on the same cohort. **(B)** A quantitative comparison of peripheral-blood lymphocyte subpopulations is presented across healthy controls (HCs), type 2 diabetes (T2DM), and T2DM-complicated tuberculosis (T2DM-TB) groups. *P < 0.05, **P < 0.01, ***P < 0.001, ****P < 0.0001.

From a total of 624 screened individuals, 198 subjects were enrolled according to the established inclusion and exclusion criteria. This cohort included 71 HCs, 67 individuals with T2DM, and 60 individuals withT2DM-TB (**Figure 1A**). The cohort exhibited a male predominance, with 69.7% of participants being male. The mean ages were 41.11 ± 12.96 years for the HCs group, 49.15 ± 7.95 years for the T2DM group, and 50.90 ± 10.43 years for the T2DM-TB group. Participants were categorized into a discovery cohort (*n* = 48) for initial omics profiling and a validation cohort (*n* = 150) for subsequent experiments. In the discovery cohort, the median glycated hemoglobin A1c (HbA1c) levels were 4.8% (IQR: 4.5–5.1%) for the HC group (*n* = 21), 7.2% (IQR: 6.8–7.5%) for the T2DM group (*n* = 17), and 7.5% (IQR: 7.2–8.1%) for the T2DM-TB group (*n* = 10).

Analysis of peripheral blood lymphocyte subsets revealed significant immune dysregulation (**Figure 1B**). Compared to HCs, patients with T2DM exhibited a notable reduction in both the frequency (P = 0.0024) and absolute count (P = 0.0137) of natural killer (NK) cells, along with an increased frequency of CD4⁺ T cells (P < 0.001). The T2DM-TB group demonstrated an additional decline in NK cells (P < 0.0001 *vs*. HC), decreased absolute counts of B cells, total T cells, and CD8⁺ T cells (all P < 0.05), and a heightened frequency of CD4⁺ T cells (P = 0.0015). A direct comparison between the T2DM-TB and T2DM groups revealed a significant increase in natural killer T (NKT) cell frequency (P = 0.0227) and marked reductions in the absolute counts of total T cells, CD4⁺ T cells, CD8⁺ T cells, and B cells (all P < 0.05), indicating severe and progressive immune dysregulation in the comorbid state.

### 3.2 Transcriptomic landscape of T2DM-TB

#### 3.2.1 Differential expression and functional enrichment of mRNAs

**Figure 2:**
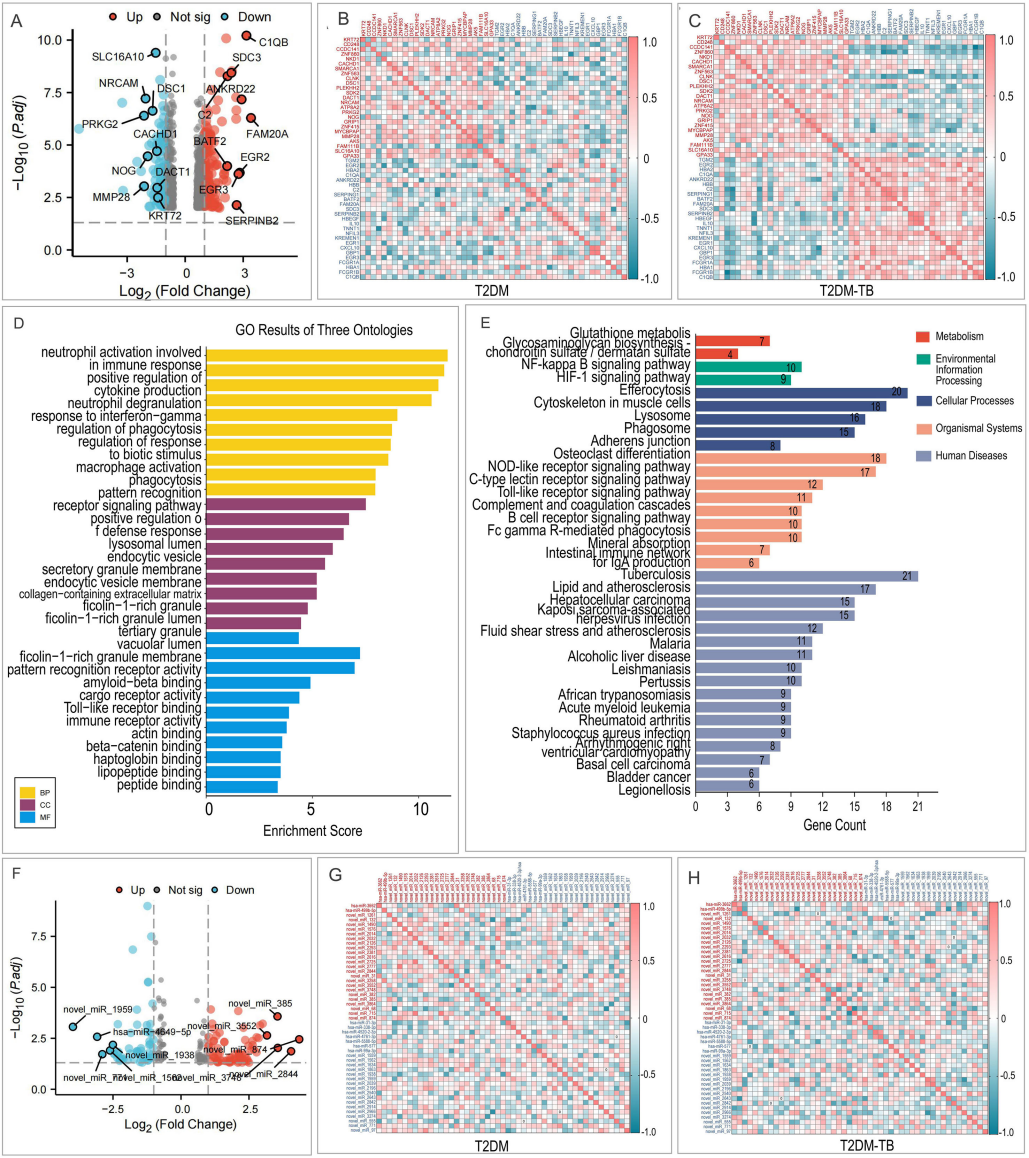
Transcriptomic landscape of T2DM-TB versus T2DM. **(A)** Volcano plot depicting differentially expressed mRNAs between T2DM-TB and T2DM groups (|log2 FC| ≥ 1.5, *P* < 0.05). **(B, C)** Correlation heatmaps of the top 25 up- and 25 down-regulated mRNAs within the T2DM **(B)** and T2DM-TB **(C)** cohorts. **(D)** GO enrichment of differential mRNAs, illustrating significant biological processes, cellular components and molecular functions. **(E)** KEGG pathway enrichment of differential mRNAs highlighting immune-metabolic cascades including Toll-like receptor, NF-κB and tuberculosis pathways. **(F)** Volcano plot of differentially expressed miRNAs (|log2 FC| ≥ 1.5, P < 0.05). **(G, H)** Correlation heatmaps of the top 25 up- and 25 down-regulated miRNAs in T2DM **(G)** and T2DM-TB **(H)**, respectively.

Whole-transcriptome sequencing of PBMCs revealed 787 DEGs between the T2DM-TB and T2DM groups (|FC| ≥ 1.5, FDR < 0.05), comprising 434 up-regulated and 353 down-regulated genes (**Figure 2A**). Notably, the highly up-regulated genes included SH2B2, RNF135, and ITGAX, which are implicated in immune modulation and cell adhesion. Conversely, down-regulated genes such as PRKG2 and NRCAM were associated with Wnt signaling and the suppression of migration. Correlation patterns exhibited notable shifts between the T2DM and T2DM-TB groups (**Figures 2B, C**). Gene Ontology (GO) enrichment analysis underscored processes including neutrophil activation, cytokine regulation, and T cell activation (**Figure 2D**, **Supplementary Table S3**). Furthermore, KEGG pathway analysis indicated significant involvement of pathways related to tuberculosis, phagocytosis, NOD-like receptor signaling, Toll-like receptor signaling, and NF-κB signaling (**Figure 2E**, **Supplementary Table S4**).

#### 3.2.2 Non-coding RNA profiles and ceRNA network construction

We identified 216 differentially expressed miRNAs, comprising 115 that were up-regulated and 101 that were down-regulated (**Figure 2F**). The most significantly up-regulated miRNAs included hsa-miR-574-3p, hsa-miR-1303, and hsa-miR-4454, whereas the most notably down-regulated miRNAs were hsa-miR-31-5p, hsa-miR-874-3p, and hsa-miR-664a-3p. Correlation analysis revealed distinct patterns between the groups, with some novel miRNAs in the T2DM group exhibiting positive correlations that shifted to negative correlations in the T2DM-TB group (**Figuress 2G, H**).

**Figure 3:**
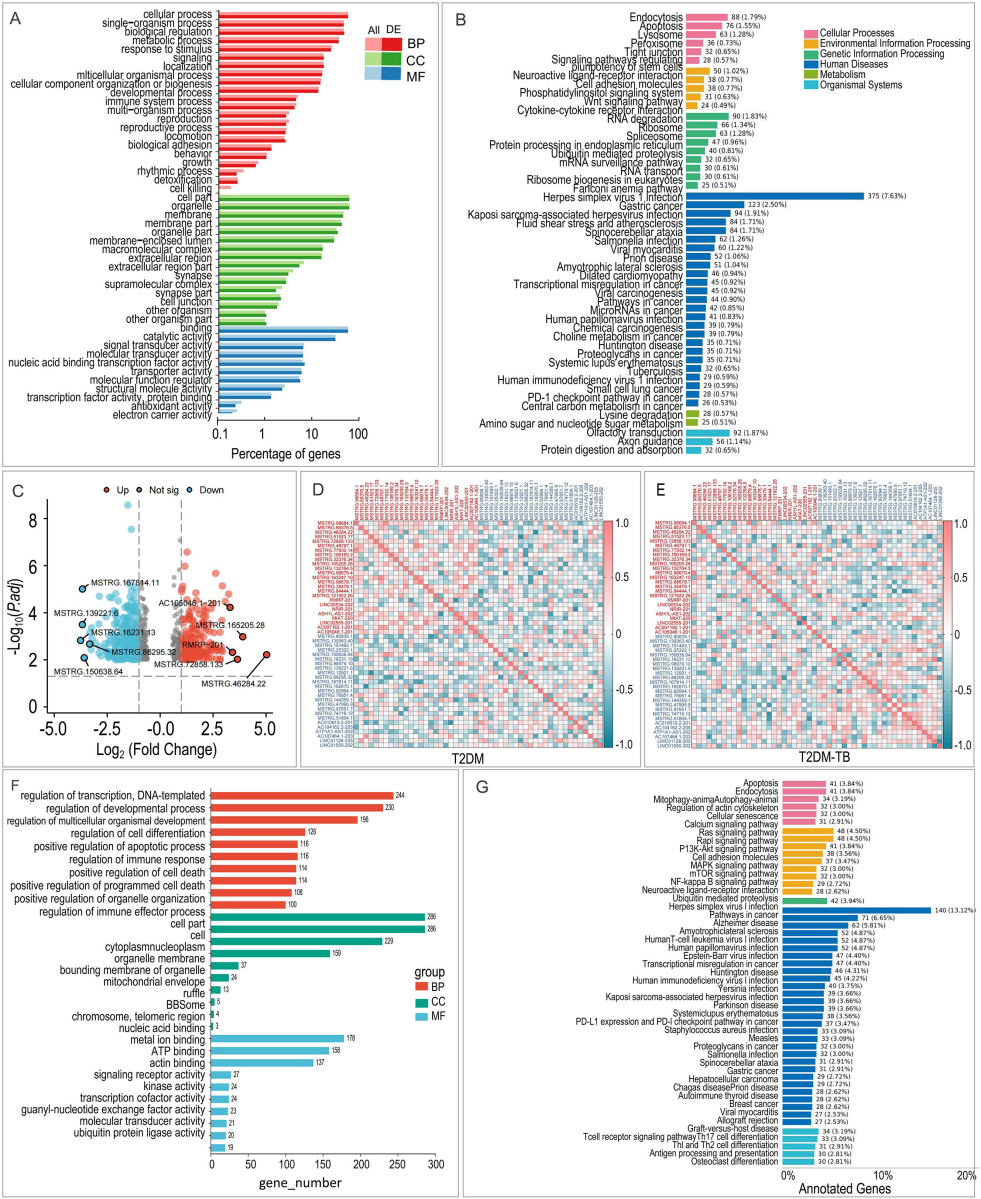
Functional landscape of differentially expressed miRNAs and lncRNAs in T2DM-TB versus T2DM. **(A, B)** GO and KEGG enrichment profiles of differential miRNAs, highlighting immune regulation, metabolic reprogramming and key signaling cascades. **(C)** Volcano plot of differential lncRNAs (|log2 FC| ≥ 1.5, *P* < 0.05). **(D, E)** Correlation heatmaps of the top 25 up- and 25 down-regulated lncRNAs within T2DM **(D)** and T2DM-TB **(E)**. **(F, G)** GO and KEGG enrichment analyses of differential lncRNAs, underscoring transcriptional control, immune modulation and disease-relevant pathways.

Functional enrichment analysis of the miRNA target genes revealed significant participation in biological processes, including cellular processes, metabolic processes, and immune system processes (**Figure 3A**). KEGG pathway analysis suggested that these miRNAs may play a role in crucial signaling pathways, such as Wnt, MAPK, NOD-like receptor, insulin, JAK-STAT, NF-κB, and PI3K-Akt (**Figure 3B**).

A total of 669 lncRNAs were found to be differentially expressed, with 285 exhibiting upregulation and 384 showing downregulation (**Figure 3C**). Correlation heatmaps indicated that the down-regulated lncRNAs in the T2DM-TB group displayed cooperative regulatory patterns (**Figures 3D, E**). GO and KEGG enrichment analyses of their cis-target genes identified functions related to transcriptional regulation, Treg differentiation, negative regulation of immune responses, cytokine secretion, and interferon signaling (**Figure 3F**). Notably, significant pathways included T cell receptor, NF-κB, JAK-STAT, and Toll-like receptor signaling (**Figure 3G**).

**Figure 4:**
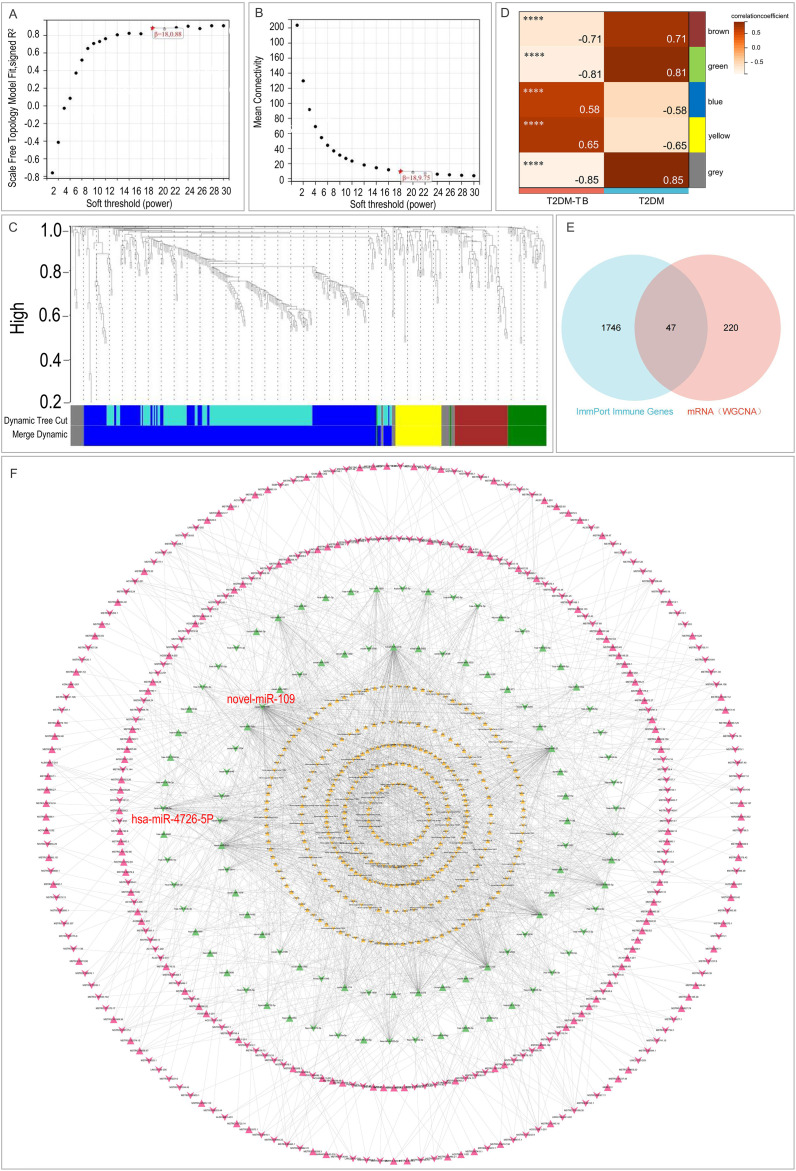
Weighted gene co-expression and ceRNA networks. **(A)** Scale-free topology fit index across soft-threshold powers. **(B)** Mean connectivity as a function of soft-threshold power. **(C)** Gene dendrogram obtained by dynamic tree cut, showing co-expression modules. **(D)** Module–trait heatmap illustrating correlations between modules and T2DM versus T2DM-TB phenotypes. **(E)** Venn diagram intersecting 267 WGCNA-derived core genes with 1–793 ImmPort immune genes, yielding 47 immune-related hub genes. **(F)** ceRNA network visualizing lncRNA–miRNA–mRNA interactions: yellow triangles = mRNAs, green triangles = miRNAs, pink triangles = lncRNAs; upward-pointing triangles denote up-regulation, downward-pointing triangles denote down-regulation. ****P < 0.0001.

WGCNA was conducted to identify gene modules significantly associated with T2DM-TB. The scale-free topology fit index and mean connectivity across various soft-thresholding powers are illustrated in **Figures 4A, B**. A soft-thresholding power (β) of 18 was chosen to achieve a scale-free topology fit index (R²) greater than 0.88. The resulting gene dendrogram and assigned modules are presented in **Figure 4C**. Module-trait relationship analysis revealed five key modules, with the blue and yellow modules exhibiting significant positive correlations with T2DM-TB (R = 0.58, P < 0.0001; R = 0.65, P < 0.0001), whereas the brown module demonstrated a strong negative correlation (R = -0.71, P < 0.0001) (**Figure 4D**). From these key modules, 267 core genes (with Module Membership > 0.8 and Gene Significance > 0.1) were identified. The intersection of these core genes with the ImmPort immune gene set resulted in 47 high-confidence, immune-related hub genes for T2DM-TB (**Figure 4E**).

To elucidate the post-transcriptional regulatory landscape, we constructed a comprehensive ceRNA network by integrating differentially expressed lncRNAs, miRNAs, and mRNAs. This network consisted of 759 nodes, including 331 lncRNAs, 328 mRNAs, and 99 miRNAs, along with 1,852 edges (**Figure 4F**). Topological analysis revealed novel-miR-109 (degree = 185) and hsa-miR-4726-5p (degree = 61) as the most connected hub miRNAs, both of which were significantly down-regulated in T2DM-TB. By concentrating on the up-regulated immune-related hub genes from the WGCNA-ImmPort intersection that were also included in the ceRNA network, we delineated two core regulatory axes: (1) MSTRG.4908.1/MSTRG.37670.90–novel-miR-109–IRF1/FPR1/LILRB3; (2) MSTRG.128052.1–hsa-miR-4726-5p–SECTM1.

This network suggests a potential mechanism where these lncRNAs act as miRNA “sponges,” sequestering novel-miR-109 and hsa-miR-4726-5p to de-repress the expression of their immune-related target mRNAs.

### 3.3 Proteomic profiling and biomarker integration

**Figure 5:**
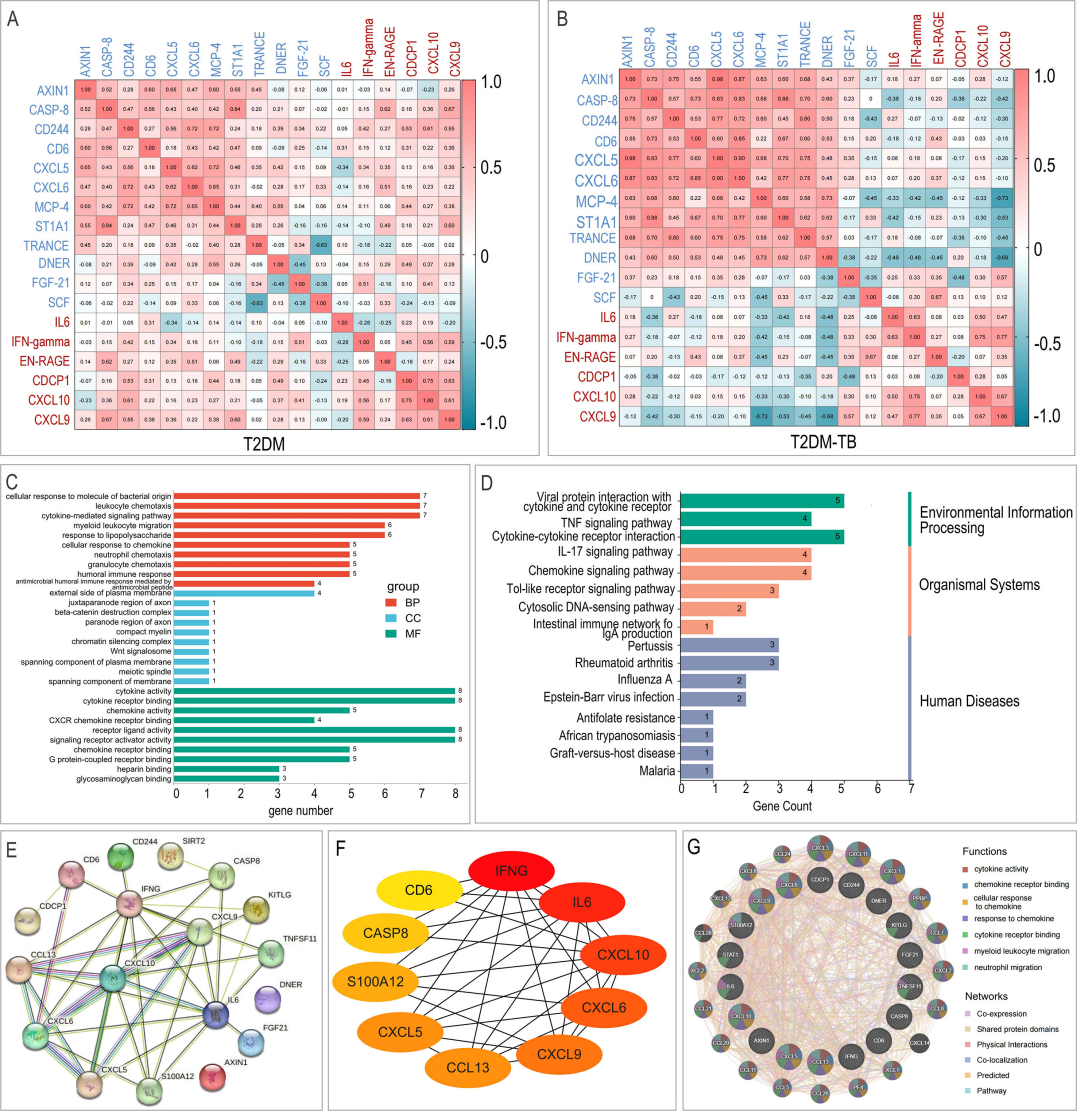
Plasma proteome landscape of T2DM-TB versus T2DM. **(A, B)** Correlation heatmaps of differential proteins within T2DM **(B)** and T2DM-TB **(C)**. **(C, D)** GO and KEGG enrichment profiles reveal immune-inflammatory cascades, cytokine signaling and pathogen-response pathways. **(E)** PPI network highlighting hub proteins and their interactions. **(F)** Top-10 high-degree hub proteins identified by Cytoscape. **(G)** GeneMANIA functional association network integrating co-expression, co-localization and pathway participation.

Plasma proteomic analysis identified 18 DEPs between T2DM-TB and T2DM (6 up, 12 down). Up-regulated proteins included IFN-γ, IL-6, CXCL9, and CXCL10, while CXCL6 and FGF-21 were among the down-regulated proteins. Correlation structures differed between groups (**Figures 5A, B**). GO and KEGG analyses emphasized functions in response to bacteria, leukocyte chemotaxis, and cytokine activity, and pathways such as viral protein-cytokine/receptor interaction and IL-17 signaling (**Figures 5C, D**). PPI network analysis identified ten hub proteins: IFNG, IL-6, CXCL10, CXCL9, CXCL6, CCL13, CXCL5, S100A12, CASP8, and CD6 (**Figures 5E, F**). GeneMANIA confirmed their functional associations through co-expression, physical interactions, and shared pathways (**Figure 5G**).

**Figure 6:**
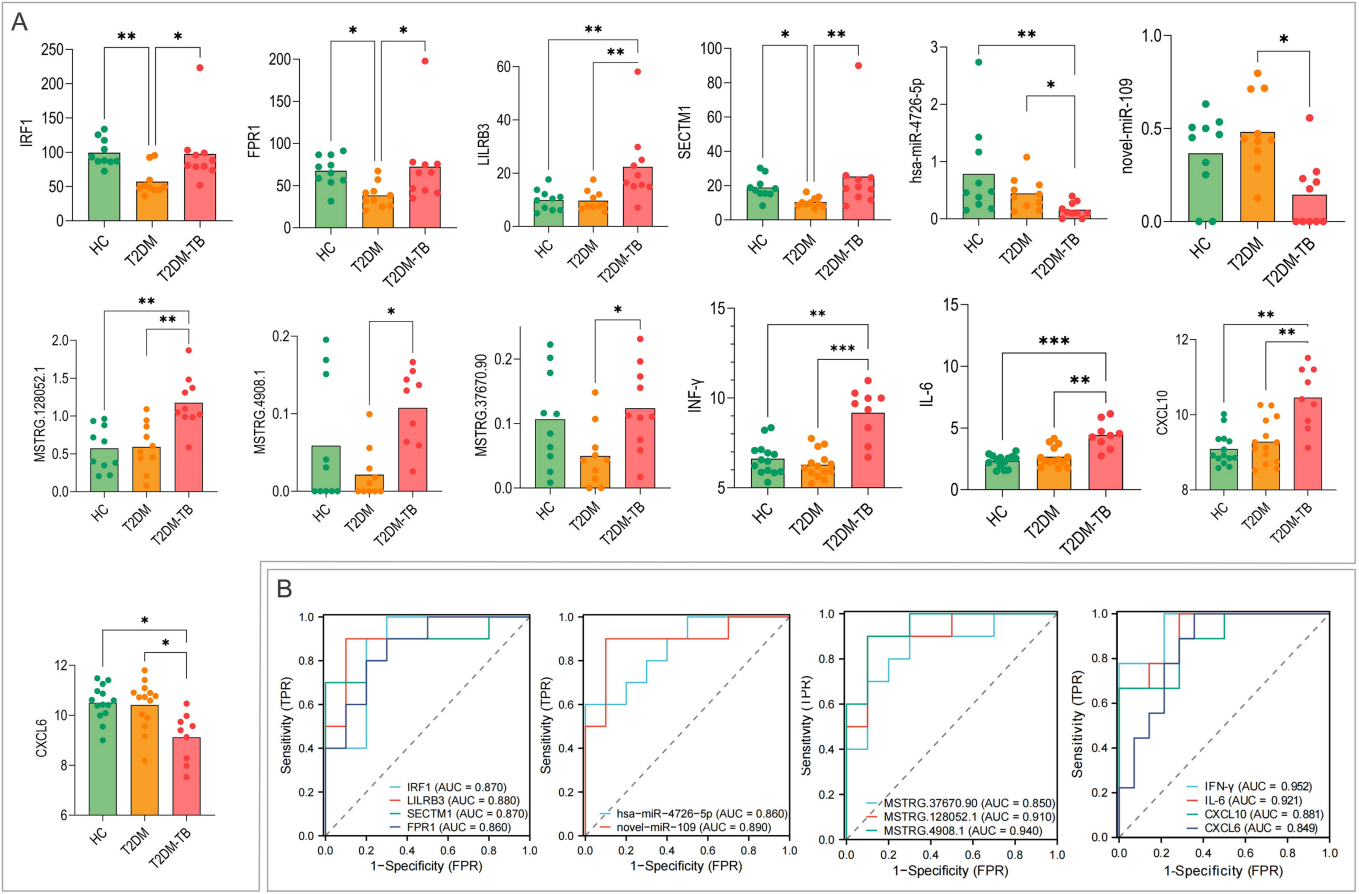
Diagnostic performance of immune-related biomarkers. **(A)** The expression levels of the 13 cross-omics biomarkers in T2DM-TB versus T2DM. **(B)** Receiver-operating-characteristic (ROC) curves demonstrating the individual and combined diagnostic efficacy of these biomarkers (AUC 0.93–0.99). *P < 0.05, **P < 0.01, ***P < 0.001.

Integrating the transcriptomic and proteomic data, and focusing on molecules within the ceRNA network, we prioritized 13 cross-platform, immune-related biomarkers for T2DM-TB: 4 mRNAs (IRF1, FPR1, LILRB3, SECTM1), 2 miRNAs (hsa-miR-4726-5p, novel-miR-109), 3 lncRNAs (MSTRG.128052.1, MSTRG.4908.1, MSTRG.37670.90), and 4 proteins (IFN-γ, IL-6, CXCL10, CXCL6). Their expression patterns are shown in **Figure 6A**. Individual ROC analysis confirmed their strong diagnostic potential, with AUC values ranging from 0.85 to 0.95 (**Figure 6B**).

### 3.4 Diagnostic model construction and validation

We constructed eleven logistic regression models using different combinations of the 13 biomarkers. The formulas for these models are as follows:


Model 1:  logit(p)=–7.515 + 0.05 × IRF1 + 0.226 × FPR1 + 0.052 × LILRB3 + 0.002 × SECTM1

Model 2: logit(p) = 4.03 – 6.323 × hsa − miR − 4726 − 5p – 7.186 × novel − miR − 109

Model 3: logit(p) = –6.278 + 16.09 × MSTRG.37670.90 + 5.678 × MSTRG.128052.1

Model 4: logit(p)=–8.039 + 5.793 × MSTRG.128052.1 + 50.087 × MSTRG.4908.1

Model 5: logit(p) = –4.635 + 0.64 × FPR1 – 8.616 × novel − miR − 109 + 51.681 × MSTRG.4908.1

Model 6: logit(p) = –1.822 + 0.262 × LILRB3 – 6.286 × novel − miR − 109

Model 7: logit(p) = –2.787 + 0.052 × FPR1 – 14.195 × novel − miR − 109 +  49.866 × MSTRG.37670.90

Model 8: logit(p) = 0.503 – 17.224 × novel − miR − 109 + 56.457 × MSTRG.37670.90

Model 9: logit(p) = –0.501 – 9.37 × novel − miR − 109 + 53.226 × MSTRG.4908.1

Model 10: logit(p) = –4.954 – 14.254 × hsa − miR − 4726 − 5p + 9.831 × MSTRG.128052.1

Model 11: logit(p) = –18.748 + 2.163 × IFN−γ + 1.995 × CXCL10 – 1.74 × CXCL6

**Figure 7:**
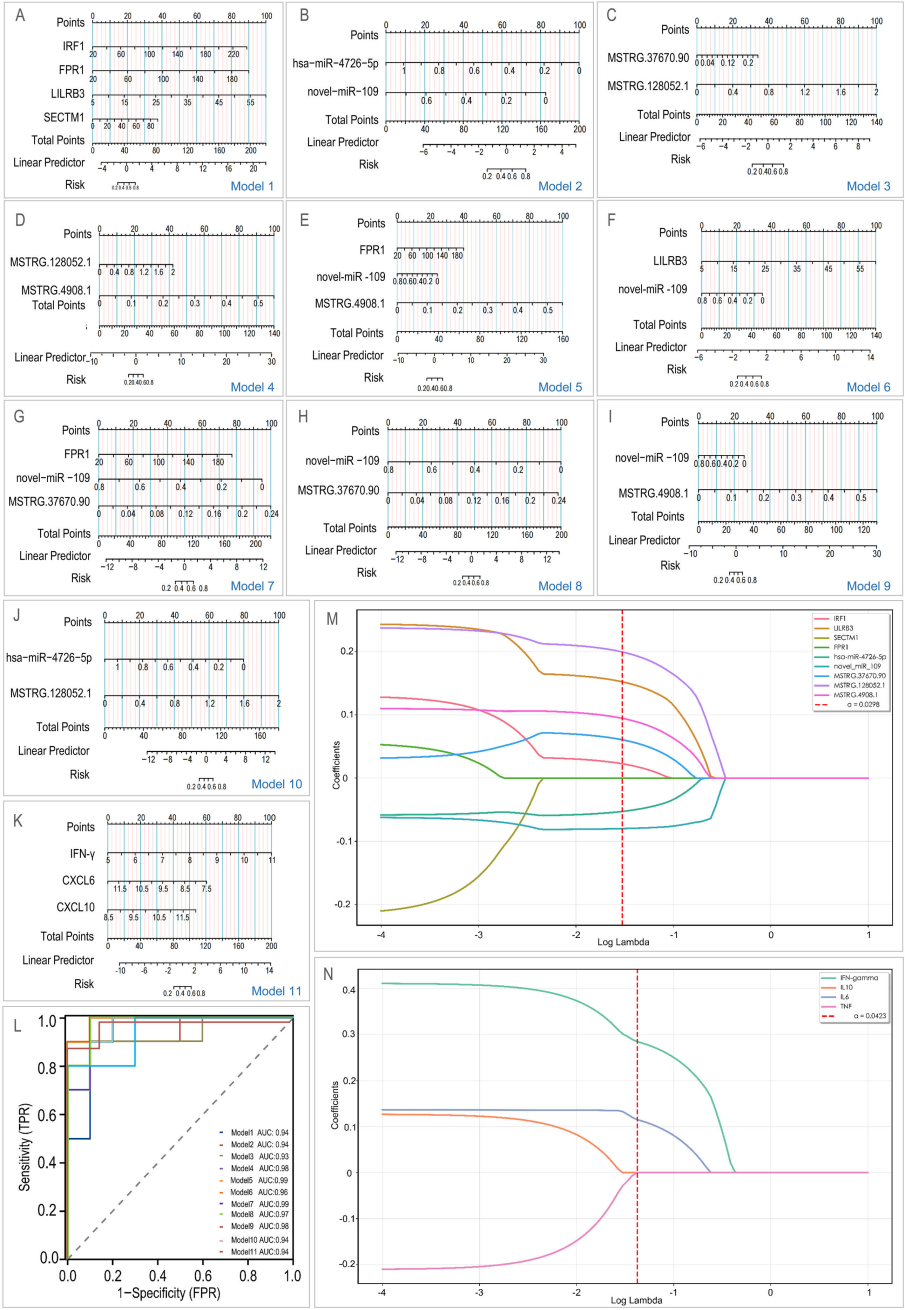
Early-warning model construction and validation. **(A–K)** Nomograms representing the 11 T2DM-TB early-warning models. **(L)** ROC curves of the 11 models, demonstrating their diagnostic efficacy (AUC 0.93–0.99). LASSO path plots: **(M)** Coefficient changes of 9 RNA features; **(N)** Coefficient changes of 4 protein features.

Nomograms for each model are illustrated in **Figures 7A–K**. All models exhibited excellent diagnostic performance in the discovery cohort, with AUC values ranging from 0.93 to 0.99 (**Figure 7L**). Model 5 (FPR1/novel-miR-109/MSTRG.4908.1) and Model 7 (FPR1/novel-miR-109/MSTRG.37670.90) attained the highest AUC of 0.99. To address the risk of overfitting due to the limited size of the discovery cohort, we conducted 5-fold cross-validation on all models, with performance metrics summarized in **Table 1**. The 11 models demonstrated stable performance, with AUC values ranging from 0.77 to 0.95, thereby confirming their robust generalization ability.

**Table 1:** Five-fold cross-validation was performed on the 11 models.

Model	Accuracy	Sensitivity	Specificity	AUC
Model1	0.75	0.70	0.80	0.77
Model2	0.85	0.90	0.80	0.86
Model3	0.75	0.70	0.80	0.85
Model4	0.90	0.90	0.90	0.83
Model5	0.85	0.80	0.90	0.88
Model6	0.90	0.90	0.90	0.83
Model7	0.75	0.70	0.80	0.85
Model8	0.85	0.90	0.80	0.88
Model9	0.85	0.90	0.80	0.90
Model10	0.85	0.90	0.80	0.92
Model11	0.8696	0.7778	0.9286	0.9524

**Figure 8:**
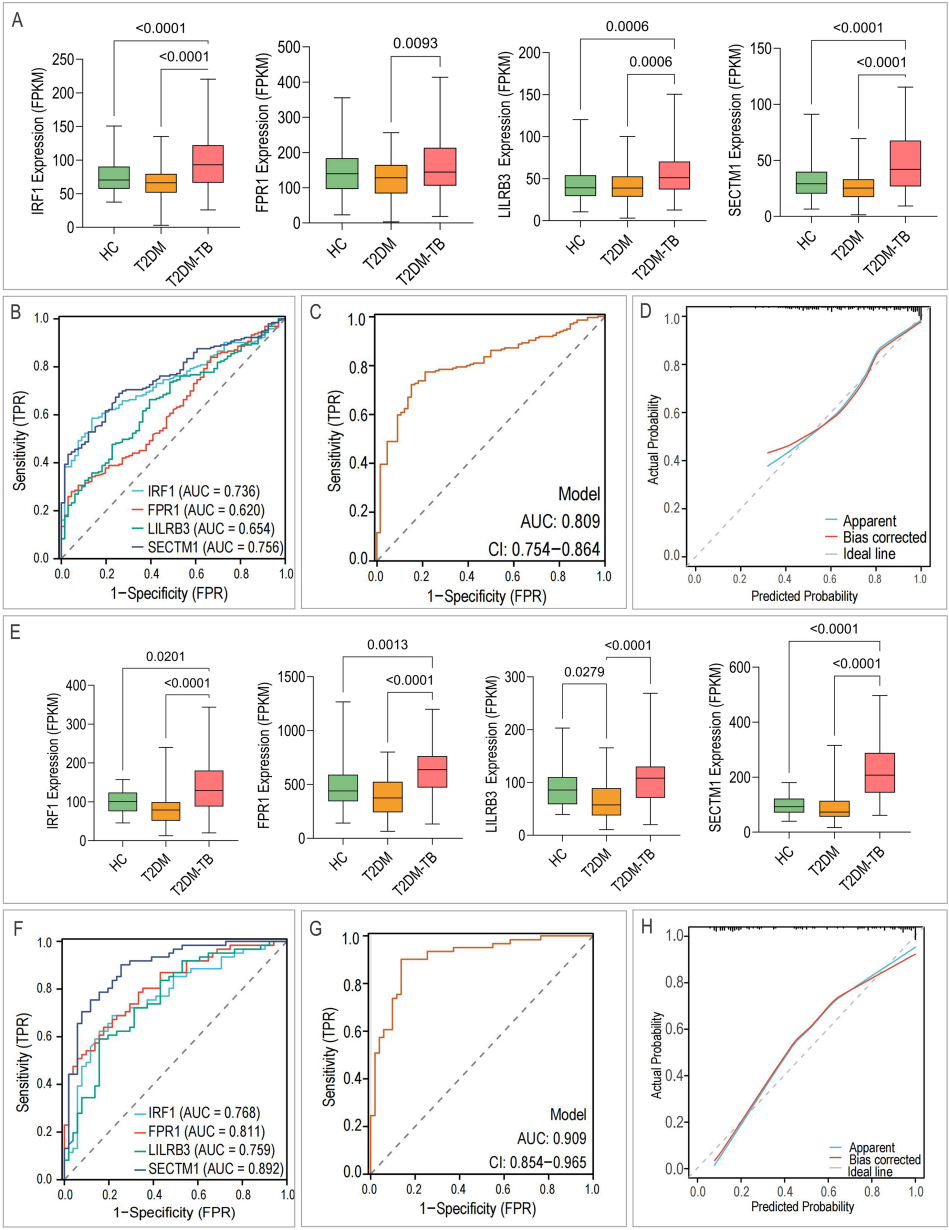
External validation of mRNA biomarkers using GEO datasets. **(A)** Differential expression of four candidate mRNAs in the GSE181143 dataset. **(B–D)** ROC curves evaluating the diagnostic efficacy of the four mRNAs in the GSE181143 dataset. **(E)** Differential expression of four candidate mRNAs in the GSE114192 dataset. **(F–H)** ROC curves evaluating the diagnostic efficacy of the four mRNAs in the GSE114192 dataset.

**Figure 9:**
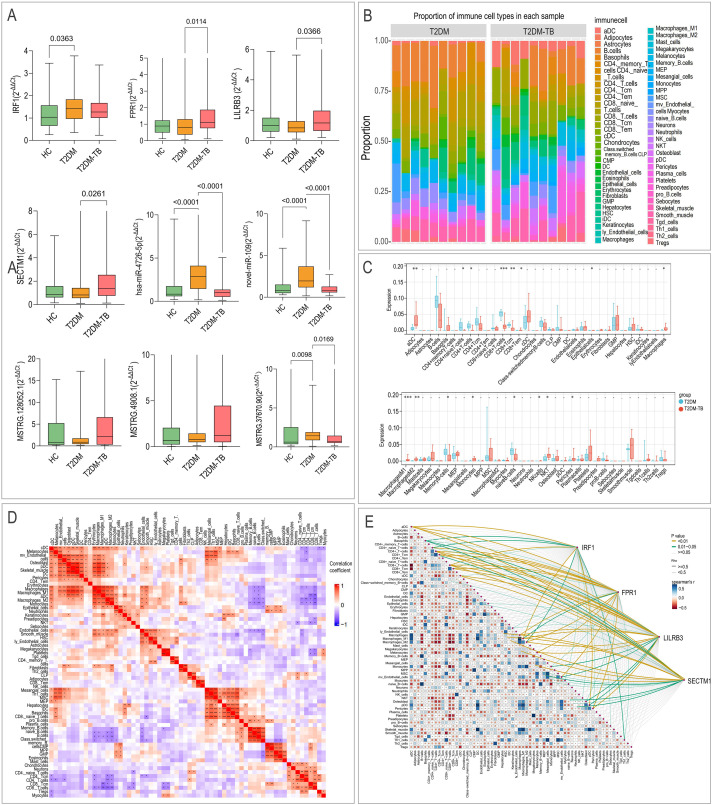
Experimental validation and immune infiltration analysis. **(A)** RT-qPCR validation of the expression levels of immune-related biomarkers (*IRF1*, *FPR1*, *LILRB3*, *SECTM1*). **(B)** xCell immune infiltration analysis comparing T2DM-TB and T2DM cohorts. **(C)** Stacked bar plot showing the relative fractions of immune cell types. **(D)** Box plot showing the differential infiltration levels of significant immune cells. **(E)** Correlation matrix of the abundance of 64 immune cell types. *P < 0.05, **P < 0.01, ***P < 0.001, ****P < 0.0001.

LASSO regression applied independently to transcriptomic and proteomic markers identified 7 of 9 RNA features (IRF1, LILRB3, hsa-miR-4726-5p, novel-miR-109, MSTRG.37670.90, MSTRG.128052.1, MSTRG.4908.1) (**Figure 7M**) and 2 of 4 proteins (IFN−γ, IL−6) (**Figure 7N**), resulting in simplified models with AUCs of 0.98 and 0.957, respectively. These core features overlapped with the most influential variables in our primary logistic models, thereby confirming their discriminative stability. External validation using GEO datasets corroborated the differential expression of the four mRNA biomarkers (IRF1, FPR1, LILRB3, SECTM1) in T2DM-TB (**Figures 8A, E**). The individual AUCs ranged from 0.620 to 0.756 (**Figure 8B**), while the combined mRNA model achieved AUCs of 0.809 (95% CI: 0.754-0.864) in GSE181143 (**Figures 8C, D**). In GSE114192, the individual AUCs ranged from 0.759 to 0.892 (**Figure 8F**) and reached 0.909 (95% CI: 0.854-0.965) (**Figures 8G, H**). RT-qPCR analysis in our validation cohort largely confirmed the expression trends of the core biomarkers, with FPR1, LILRB3, SECTM1, hsa-miR-4726-5p, and novel-miR-109 exhibiting consistent and significant changes (**Figure 9A**). However, MSTRG.4908.1 demonstrated only a non-significant increase, while MSTRG.37670.90 exhibited an opposite trend. This discrepancy may be attributed to small-sample bias, lncRNA molecular characteristics, and divergent detection principles.

### 3.5 Immune microenvironment and mechanistic insights

xCell immune infiltration analysis revealed a distinct “pro-inflammatory-suppressive-reconstructive” imbalance in T2DM-TB (**Figures 9B–D**). This was characterized by hyperactivated innate immunity (increased M1/M2 macrophages, monocytes, NKT cells) and compromised adaptive immunity (decreased CD4⁺/CD8⁺ T cells and B cells). Furthermore, an immunosuppressive milieu (elevated Tregs, Th2 cells) along with elevated gene expression signatures associated with stromal and endothelial functions.

**Figure 10:**
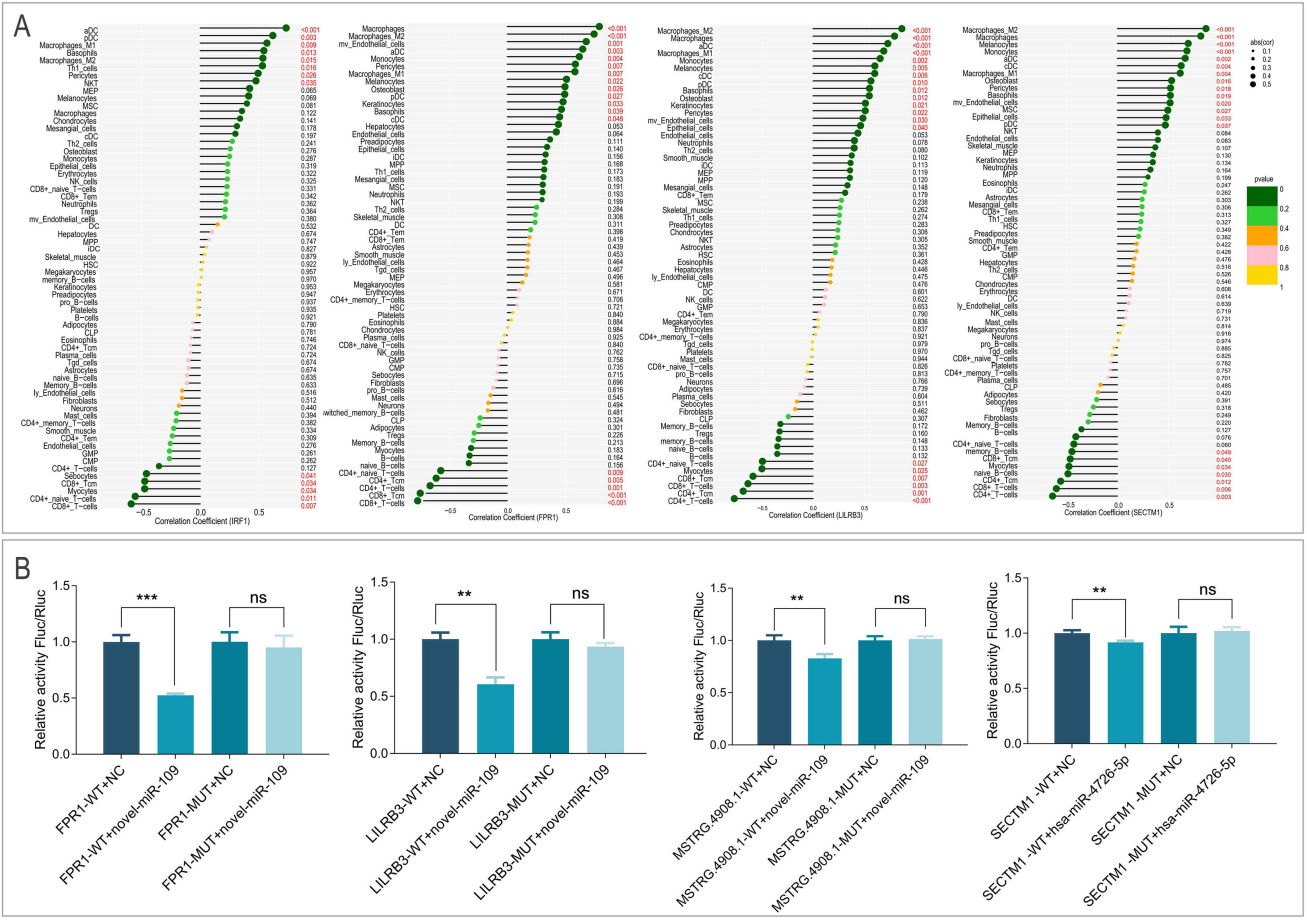
Correlation and mechanistic validation. **(A)** Correlation analysis between the expression of specific T2DM-TB immune biomarkers (IRF1, FPR1, LILRB3, SECTM1) and the abundance of specific immune cells. **(B)** Dual-luciferase reporter assays validating miRNA-mRNA binding interactions. **P < 0.01, ***P < 0.001, ns: no significance.

Correlation analysis showed that the mRNA biomarkers (IRF1, FPR1, LILRB3, SECTM1) were positively associated with innate immune cells (M1/M2 macrophages, monocytes, NKT cells) and negatively associated with adaptive immune subsets (CD4⁺/CD8⁺ T cells, B cells) (**Figures 9E**, **10A**). Finally, dual-luciferase reporter assays confirmed that novel-miR-109 directly binds to the wild-type sites in FPR1, LILRB3, and MSTRG.4908.1 (all P < 0.05), and hsa-miR-4726-5p targets the SECTM1 3’UTR (P < 0.05), as mutations in these sites abolished the binding (**Figure 10B**).

## 4 Discussion

T2DM is one of the strongest independent risk factors for TB (14). When the two diseases coexist, they amplify each other’s pathogenicity, leading to more severe clinical manifestations, enhanced drug resistance, higher relapse rates, and increased mortality (5, 11, 34, 43). Early detection of incident TB among individuals with T2DM therefore represents an urgent unmet clinical need. By integrating whole-transcriptome and plasma-proteome data, we have generated eleven discriminatory models that achieve AUCs of 0.93–0.99—performances that exceed existing reports. Specifically, our models outperform breath-volatile signatures (AUC = 0.92) (44) and clinical-parameter classifiers (AUC = 0.78–0.82) (45, 46) by 7.6% to 21%, and they retain excellent generalizability in two independent GEO cohorts (AUC = 0.809–0.909). RT-qPCR validation of seven core molecules (e.g., FPR1, novel-miR-109, MSTRG.4908.1) confirmed directional concordance with sequencing data. Unlike prognostic constructs (47) or purely mechanistic multi-omics studies (48), our models are explicitly designed for early differential diagnosis, thereby offering clinicians a substantially widened intervention window.

The models demonstrated high diagnostic performance (AUC 0.93–0.99) and stable generalization during 5-fold cross-validation (AUC 0.77–0.95). While some regression coefficients were substantial, possibly due to multicollinearity or scaling, LASSO regression verified that a core subset of features (e.g., IRF1, novel-miR-109, IFN-γ) maintained strong predictive performance (AUC > 0.95), thereby supporting the overall reliability of our biomarker signatures. For future applications, a simplified model based on LASSO-selected features may improve generalizability (49, 50).

The eleven classifiers are built on thirteen immune-related biomarkers that capture the cross-omics dysregulation characteristic of T2DM-TB. These include the previously unreported non-coding RNAs novel-miR-109 (AUC = 0.89) and lncRNA MSTRG.4908.1 (AUC = 0.94), together with the high-specificity proteins IFN-γ (AUC = 0.952). Functionally, the biomarkers operate within a multi-layer network that integrates innate activation, adaptive exhaustion and metabolic reprogramming: (i) IRF1, a master interferon-responsive transcription factor, augments macrophage microbicidal capacity via IFN-γ but, when chronically elevated, fuels oxidative injury and tissue damage (51, 52). (ii) FPR1 orchestrates phagocyte chemotaxis; its over-activation intensifies MAPK-mediated inflammation in T2DM-TB (53, 54). (iii) LILRB3 delivers inhibitory signals that blunt antigen presentation and constrain T-cell priming (55). (iv) SECTM1, whose levels correlate with sputum bacillary load (56, 57), promotes activation of T cells and monocytes. (v) Non-coding RNAs (novel-miR-109, lncRNAs) modulate monocyte–macrophage trafficking and immunosuppressive circuits through competing endogenous RNA (ceRNA) mechanisms (58–60). (vi) Inflammatory cytokines IFN-γ, IL-6, CXCL10 and CXCL6 perpetuate chronic inflammation and parenchymal destruction (61–64). Compared with single-omics signatures (65, 66) or treatment-response models (48, 67), our biomarker panel offers superior specificity for early screening.

By integrating flow cytometry with in-silico immune-infiltration analyses, we delineate a triphasic microenvironmental signature—”inflame–suppress–remodel”—in T2DM-TB. Compared to T2DM alone, patients exhibit hyper-activated innate immunity, characterized by increased M1/M2 macrophages, monocytes, aDC, and NKT cells (all P < 0.05), alongside progressive adaptive immune impairment, evidenced by reduced CD4⁺ and CD8⁺ T cells and B cells (P < 0.001–0.01). The central molecular axis driving this imbalance appears to be IRF1-mediated macrophage over-activation (R = 0.82), which amplifies IL-6/TNF-α cascades (68) while simultaneously repressing T-bet-dependent Th1 differentiation (51, 52, 69, 70). Notably, elevated systemic IFN-γ, driven by M1 macrophages/NK/NKT cells (xCell: P < 0.05), coexists with Th2-biased tolerance, as indicated by increased Th2 cells, IL-4, and IL-13 (P < 0.05). This creates a “hyperinflammation-hyporesponsiveness” paradox, wherein innate IFN-γ amplifies local inflammation, while Th2/Treg (P < 0.01)-LILRB3-mediated suppression impairs bacterial clearance. FPR1-facilitated monocytic chemotaxis and endothelial remodeling, mediated by CXCL8/CCL2 and VEGF signals, sustain granuloma formation (71) but also promote CD8⁺ T-cell dysfunction through PD-L1/CTLA-4 signaling (69). LILRB3, by engaging MHC-I and activating SHP-1 phosphatase (55), collaborates with SECTM1-linked B-cell networks to suppress class-switch recombination, as indicated by down-regulated AID (72). Furthermore, skewed NKT-cell activation (IL-4/IL-13) synergizes with M2 macrophages (73, 74) and SECTM1-expressing endothelial cells to reinforce a Th2-biased, immune-tolerant milieu (71, 75, 76). Elevated gene expression patterns indicative of fibroblast and endothelial activity were also observed (P < 0.01), potentially reflecting systemic immune–stromal communication and contributing to profibrotic pathways, such as CXCL10-driven fibrosis in affected tissues (77–79).

Convergent pathway analysis demonstrated enrichment in immune response regulation, inflammatory signaling, Toll-like receptor, NOD-like receptor, and NF-κB cascades (70, 80, 81). Additionally, JAK-STAT, PI3K-Akt, and MAPK networks play critical roles in modulating leukocyte survival and differentiation (82). The Wnt, Ras, and HIF-1 pathways indicate metabolic reprogramming and immune-metabolic crosstalk (77–79, 83). In contrast, p53 signaling may undermine host defense by influencing cell-cycle checkpoints and apoptosis (84).

This study possesses considerable clinical translational value. At the diagnostic level, the combined detection of gene and protein biomarkers can simultaneously evaluate infection activity and the severity of metabolic disorders, surpassing the single-respiratory volatilomics model proposed by Xu et al. (44). At the therapeutic level, LILRB3 inhibitors or novel-miR-109 agonists may reverse immune suppression, while FPR1 antagonists, such as Cyclosporin H, may mitigate excessive inflammation. These findings offer new strategies for the concurrent management of infection and metabolic disorders. Future research should focus on advancing precision interventions by targeting pro-inflammatory immune regulation, restoring adaptive immunity, and reversing immune tolerance.

This study presents several limitations: (1) The relatively small sample size of the discovery cohort may compromise the generalizability of the results and the statistical power. (2) The analyses of the biological functions of the 13 potential biomarkers were confined to the molecular level, lacking comprehensive investigations using animal models. (3) This study predominantly relies on cross-sectional data, which does not capture the dynamic changes in immune markers among T2DM-TB patients, and it did not stratify by key immune cell subsets to investigate disease heterogeneity. The validation of biomarkers was conducted solely through GEO datasets and RT-qPCR, while miRNA, lncRNA, and protein biomarkers lack independent external validation and necessitate further verification. (4) Although early risk warning models were developed, their clinical translational potential has not been assessed in real-world clinical environments. (5) Discrepancies in expression trends of certain lncRNAs were noted between transcriptome sequencing and RT-qPCR validation, which may primarily result from small-sample bias, intrinsic molecular characteristics of lncRNAs, and limitations inherent in lncRNA detection technologies. Future research should aim to increase sample sizes, refine data analysis methods, conduct longitudinal studies, perform prospective clinical trials, stratify cohorts by HbA1c to examine the influence of long-term glycemic control on immune characteristics and model performance, and stratify the T2DM-TB group by key immune cell subsets to investigate variations in biomarker expression, disease severity, and treatment response among subgroups. These efforts are essential to address the identified limitations and facilitate the translation of findings into clinical applications.

## 5 Conclusions

This integrated multi-omics study identified 13 immune-related biomarkers and developed 11 early-warning models for T2DM-TB, achieving a high predictive accuracy (AUC 0.93–0.99). Key biomarkers, including IRF1, FPR1, novel-miR-109, and MSTRG.4908.1, function within a ceRNA network that promotes a pro-inflammatory yet immunosuppressive microenvironment. Additionally, we uncovered a triad of immune dysfunction characterized by hyperactivated innate immunity, diminished adaptive responses, and increased stromal-associated gene expression, which drives T2DM-TB progression. These findings provide novel insights into molecular mechanisms and present clinically translatable tools for early detection and precision intervention.

## Data Availability

All data generated or analyzed during this study are included in this published article and its **Supplementary Material**. If necessary, data can be reasonably requested from the corresponding author.

## Supplementary material

Supplementary Figure 1 pmirGLO vector map.
