# Supplementary figures and images for: Integrated multi-omics profiling reveals immune-related biomarkers and regulatory networks for early prediction of tuberculosis in type 2 diabetes mellitus
Source: Front Immunol. 2026 Feb 26;17:1755184. doi: 10.3389/fimmu.2026.1755184 (PMC12979386; doi:10.3389/fimmu.2026.1755184)

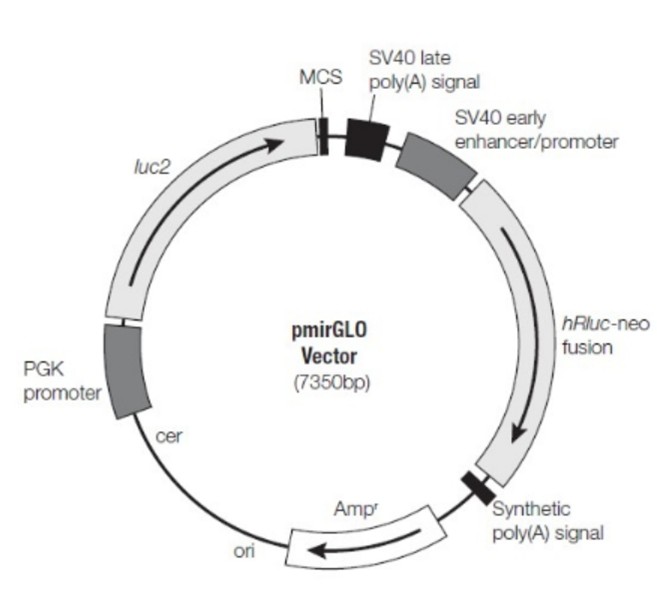

Supplement: Supplementary Figure 1 — pmirGLO vector map. [file Image1.jpeg]
