# Supplementary material for: Integrated multi-omics profiling reveals immune-related biomarkers and regulatory networks for early prediction of tuberculosis in type 2 diabetes mellitus
Source: Front Immunol. 2026 Feb 26;17:1755184. doi: 10.3389/fimmu.2026.1755184 (PMC12979386; doi:10.3389/fimmu.2026.1755184)
Supplement: Supplementary file 2 [file Table1.docx]

**Table S1.** Details of GEO Data.

| **Year** | **Accession** | **Platform** | **Sequencing type** | **Sample (n)** | | **Sample source country** | **Species** | **Tissue** |
| --- | --- | --- | --- | --- | --- | --- | --- | --- |
| 2021 | GSE181143 | [GPL20795](https://www.ncbi.nlm.nih.gov/geo/query/acc.cgi?acc=GPL20795) | RNA seq | 348 ( DM-TB: 193，  DM: 66, HC: 89 ) | | Salvador, Brazil, Pune, India | Homo sapiens | Whole blood |
| 2020 | GSE114192 | GPL18573 | RNA seq | 149 (DM-TB: 61,  DM: 52, HC: 36) | Romania, Indonesia, South Africa, Peru | | Homo sapiens | Whole blood |

Abbreviations: DM, Diabetes Mellitus; DM-TB, Diabetes Mellitus with Tuberculosis; GEO, Gene Expression Omnibus; HC, Healthy Controls;

TB, Tuberculosis.
