# Supplementary material for: Integrated multi-omics profiling reveals immune-related biomarkers and regulatory networks for early prediction of tuberculosis in type 2 diabetes mellitus
Source: Front Immunol. 2026 Feb 26;17:1755184. doi: 10.3389/fimmu.2026.1755184 (PMC12979386; doi:10.3389/fimmu.2026.1755184)
Supplement: Supplementary file 3 [file Table2.docx]

**Table S2. Detailed information on primer sequence of each gene symbol and internal reference gene.**

| **Gene symbol** | **Primer Sequence** |
| --- | --- |
| IRF1 | Forward: 5′-CTGTGCGAGTGTACCGGATG-3′ |
|  | Reverse: 5′ATCCCCACATGACTTCCTCTT-3′ |
| FPR1 | Forward: 5′-CACACAGTCACCACCATCAGTTACC-3′ |
|  | Reverse: 5′GCACAGGAACCAGCCGAAAGG-3′ |
| LILRB3 | Forward: 5′-GTCTGGGAAGATACCTGGAGG-3′ |
|  | Reverse: 5′GGACGCTGGAAATCAGTCTTT-3′ |
| SECTM1 | Forward: 5′-GGGACACCAGAGAAATAACAGACAAG-3′ |
|  | Reverse: 5′AGAGCGACCAAGAGGATGAAGAC-3′ |
| hsa-miR-4726-5p | Forward: 5′-TATTATTAGGGCCAGAGGAGCCTGGA-3′ |
| novel-miR-109 | Forward: 5′-TATATAACAGGCTGGAGTGCTGTGGCA-3′ |
| MSTRG.128052.1 | Forward: 5′-ATCTTGACTCACTGCTACCTCCATC-3′ |
|  | Reverse: 5′AAGTTGGCTGGGCGTGGTG-3′ |
| MSTRG.4908.1 | Forward: 5′-AAACAGGAGGCTGCCATGAT-3′ |
|  | Reverse: 5′TCCTGCTGATCCTGACTTGC-3′ |
| MSTRG.37670.90 | Forward: 5′-TGAGTTGCATTCCAGCTTCCT-3′ |
|  | Reverse: 5′TGGCTTACGTGACTAGGCTG-3′ |
| GAPDH | Forward: 5′-CTCTGGTAAAGTGGATATTGT-3′ |
|  | Reverse: 5′-GGTGGAATCATATTGGAACA-3′ |
