# Supplementary material for: Integrated multi-omics profiling reveals immune-related biomarkers and regulatory networks for early prediction of tuberculosis in type 2 diabetes mellitus
Source: Front Immunol. 2026 Feb 26;17:1755184. doi: 10.3389/fimmu.2026.1755184 (PMC12979386; doi:10.3389/fimmu.2026.1755184)
Supplement: Supplementary file 4 [file Table3.docx]

Table S3. Top10 entries for GO enrichment of differential mRNAs in T2DM-TB vs T2DM groups.

| ID | Term | Count ^a^ | *P* adjust | Genes |
| --- | --- | --- | --- | --- |
| BP |  |  |  |  |
| GO:0002283 | neutrophil activation involved in immune response | 51/664 | 1.51195E-08 | CTSA; NPC2; OSCAR; FCGR2A; ASAH1; CD93; TNFRSF1B; S100A11; SLC11A1; HBB; PYGL; RAB31; FGR; LILRB2; FCN1; CTSS; FPR1; DSC1; BST1; HK3; NFAM1; CTSB; NEU1; LILRA2; LILRB3; GM2A; FPR2; FCER1G; MGST1; DOK3; ITGAX; HPSE; FTH1; CRISPLD2; GNS; SERPINA1; FGL2; ANPEP; LTA4H; CD36; PLEKHO2; S100A9; CD63; PGRMC1; TLR2; CYBB; HSPA6; SIRPA; CD14; CREG1; TIMP2 |
| GO:0043312 | neutrophil degranulation | 50/664 | 1.89637E-08 | CTSA; NPC2; OSCAR; FCGR2A; ASAH1; CD93; TNFRSF1B; S100A11; SLC11A1; HBB; PYGL; RAB31; FGR; LILRB2; FCN1; CTSS; FPR1; DSC1; BST1; HK3; NFAM1; CTSB; NEU1; LILRB3; GM2A; FPR2; FCER1G; MGST1; DOK3; ITGAX; HPSE; FTH1; CRISPLD2; GNS; SERPINA1; FGL2; ANPEP; LTA4H; CD36; PLEKHO2; S100A9; CD63; PGRMC1; TLR2; CYBB; HSPA6; SIRPA; CD14; CREG1; TIMP2 |
| GO:0001819 | positive regulation of cytokine production | 48/664 | 1.51195E-08 | STAT1; TRIM16; IRF1; FZD5; SLC11A1; RIPK2; FGR; TXK; LILRA5; LILRB2; TRPV4; FCN1; IL15; CLNK; TLR8; NFAM1; CD86; SEC14L1; CCR7; LILRA2; MYD88; CLU; SCIMP; CLEC4E; IL17RC; SRC; FCER1G; IL23A; TLR1; HIF1A; RNF135; IL10; GBP5; HPSE; TLR7; EGR1; LILRB1; F2RL1; FFAR2; AIF1; IRF7; TLR4; CD36; CARD9; TLR2; SPHK1; BCL3; CD14 |
| GO:0002831 | regulation of response to biotic stimulus | 41/664 | 1.41154E-06 | SUSD4; IFNGR2; STAT1; IRF1; CD209; RIPK2; FGR; TXK; SERPING1; FCN1; IL15; CLNK; CD1D; SASH1; TLR8; TRIB1; SEC14L1; PPARG; LILRA2; SCIMP; FPR2; CLEC4E; SRC; FCER1G; NECTIN2; IL23A; PARP9; RNF135; GBP5; PLSCR1; SLAMF8; LILRB1; F2RL1; FFAR2; IRF7; FGL2; TLR4; CD36; CARD9; HCK; HMGB3 |
| GO:0006909 | phagocytosis | 39/664 | 1.62674E-06 | DYSF; FCGR2A; CD93; TGM2; SLC11A1; RAB31; FGR; C2; FCN1; IL15; MSR1; NCF2; CD302; MARCO; PPARG; MYD88; FPR2; SRC; FCER1G; GAS6; ABCA1; HAVCR1; DOCK1; MYO7A; F2RL1; IL15RA; AIF1; TLR4; FCGR1A; RAB34; CD36; MERTK; MFGE8; TLR2; HCK; SPHK1; SIRPA; CD14; MYO10 |
| GO:0050727 | regulation of inflammatory response | 39/664 | 1.54397E-05 | PIK3AP1; TGM2; NAPEPLD; TNFRSF1B; MAPK7; LILRA5; TRPV4; IL15; NT5E; BST1; CCR7; PPARG; MYD88; GPX1; FPR2; IL17RC; MEFV; FCER1G; IL23A; SBNO2; BCL6; LRRK2; IL10; GBP5; SLAMF8; TLR7; PLA2G7; FFAR2; LACC1; TLR4; S100A9; CNR2; TLR2; HCK; SPHK1; CASP5; SNCA; SETD6; SIRPA |
| GO:0042110 | T cell activation | 39/664 | 0.000188841 | DUSP3; CTSL; IRF1; TNFRSF1B; FZD5; ICAM1; SLC11A1; TNFSF13B; CD209; RIPK2; VCAM1; TESPA1; LILRB2; IL15; TCF7; CD1D; LEF1; CD86; CCR7; BTLA; CDH26; CLEC4E; SRC; FCER1G; IL23A; CAMK4; BCL6; IL10; HLX; EGR1; LILRB1; F2RL1; AIF1; FGL2; EGR3; BCL3; CD151; LILRB4; SIRPA |
| GO:0031349 | positive regulation of defense response | 38/664 | 5.27465E-06 | TGM2; NAPEPLD; CD209; RIPK2; TXK; LILRA5; TRPV4; FCN1; IL15; CLNK; CD1D; TLR8; CCR7; LILRA2; MYD88; FPR2; CLEC4E; IL17RC; SRC; FCER1G; NECTIN2; IL23A; PARP9; LRRK2; GBP5; PLSCR1; TLR7; PLA2G7; F2RL1; FFAR2; IRF7; TLR4; CARD9; S100A9; TLR2; HCK; HMGB3; SNCA |
| GO:0050867 | positive regulation of cell activation | 37/664 | 6.43991E-05 | TNFSF13B; CD209; RIPK2; FGR; VCAM1; TESPA1; LILRA5; LILRB2; IL15; CD1D; LEF1; BST1; CD86; CCR7; LILRA2; BTLA; SRC; FCER1G; NECTIN2; IL23A; GAS6; BCL6; HAVCR1; LRRK2; IL10; HLX; T2DMTN; LILRB1; F2RL1; AIF1; EGR3; TLR4; PLEK; MMP14; GAB2; LILRB4; SIRPA |
| GO:0030099 | myeloid cell differentiation | 37/664 | 9.87321E-05 | IL31RA; STAT1; OSCAR; MYL9; IL15; BATF2; LEF1; SNX10; TRIB1; TRIM58; CCR7; CCR1; PPARG; LILRB3; SRC; FCER1G; IL23A; SBNO2; CAMK4; BCL6; HIF1A; T2DMTN; NFE2; LILRB1; F2RL1; FAM20C; IRF7; SPI1; ZNF385A; TLR4; MPIG6B; ITGA2B; GAB2; TLR2; HMGB3; LILRB4; LTBR |
| CC |  |  |  |  |
| GO:0062023 | collagen-containing extracellular matrix | 35/679 | 0.000221719 | CTSL; LTBP3; TGM2; VCAN; COL6A3; ICAM1; NAV2; F13A1; C1QA; IMPG2; SERPING1; FCN1; CTSS; ADAMTSL4; TECTA; HSPG2; CTSF; EMILIN2; CTSB; SDC3; CLU; SDC2; MMP28; TIMP1; PLSCR1; SERPINA1; FGL2; S100A9; MFGE8; ANXA5; CD151; SPARC; MEGF9; C1QB; TIMP2 |
| GO:0030139 | endocytic vesicle | 31/679 | 3.75535E-05 | DYSF; FZD5; HBA2; SLC11A1; HBB; RAB31; CTSS; MSR1; NCF2; RILP; MARCO; SCIMP; TLR1; HBEGF; MCOLN1; ABCA1; WNT7A; TLR7; STAB1; FCGR1A; RAB34; CD36; CD163; SCARF1; HBA1; TLR2; SPHK1; RAB32; CYBB; FCGR1B; SPARC |
| GO:0009897 | external side of plasma membrane | 31/679 | 0.002461204 | CD248; IL31RA; ICAM1; CD209; VCAM1; FCN1; CD1D; MSR1; TLR8; CD86; CD302; CTSB; ADAM9; CCR7; CCR1; NRCAM; FCER1G; ABCA1; S1PR1; LILRB1; ABCC4; CXCL10; TLR4; ANPEP; ITGA2B; CD36; MFGE8; CD163; ANXA5; CCRL2; CD14 |
| GO:0030667 | secretory granule membrane | 30/679 | 4.92303E-05 | FCGR2A; CD93; TNFRSF1B; SLC11A1; RAB31; LILRB2; FPR1; DSC1; BST1; NFAM1; LILRB3; FPR2; ACRBP; FCER1G; MGST1; DOK3; ITGAX; ABCC4; ANPEP; ITGA2B; CD36; CD63; PGRMC1; TLR2; SNCA; CYBB; SPARC; LAMP3; SIRPA; CD14 |
| GO:0031252 | cell leading edge | 28/679 | 0.01389579 | DYSF; PTPRK; S100A11; CARMIL1; FGR; ATP6V1B2; SRA1; TRPV4; CDC42BPA; EPB41L3; APBB1; PLEKHH2; WASF1; SCIMP; THEM4; SRC; MEFV; MCC; ABLIM1; ADGRE2; KANK1; AIF1; TLR4; PLEK; EPB41L5; PDE9A; CTNNA1; MYO10 |
| GO:0005925 | focal adhesion | 27/679 | 0.021864285 | STARD8; RHOU; TGM2; ICAM1; PGM5; LAP3; TRPV4; HSPG2; TPM4; PRAG1; MARCKS; ADAM9; WASF1; PEAK3; ABCB4; TLE2; SRC; NECTIN2; ITGA2B; EPB41L5; MMP14; HCK; ANXA5; CD151; FZD1; RPS5; CTNNA1 |
| GO:0030055 | cell-substrate junction | 27/679 | 0.026814553 | STARD8; RHOU; TGM2; ICAM1; PGM5; LAP3; TRPV4; HSPG2; TPM4; PRAG1; MARCKS; ADAM9; WASF1; PEAK3; ABCB4; TLE2; SRC; NECTIN2; ITGA2B; EPB41L5; MMP14; HCK; ANXA5; CD151; FZD1; RPS5; CTNNA1 |
| GO:0098857 | membrane microdomain | 26/679 | 0.00341462 | DYSF; MYOF; TNFRSF1B; RGMB; ICAM1; NFAM1; ABCB4; SRC; MAL; TLR1; ATP1B1; ABCA1; S1PR1; GRIP1; LRRK2; HPSE; PLSCR1; AKAP6; CD36; PSEN2; TLR2; HCK; ATP1B3; CTNNA1; CD14; LRP6 |
| GO:0098589 | membrane region | 26/679 | 0.005448366 | DYSF; MYOF; TNFRSF1B; RGMB; ICAM1; NFAM1; ABCB4; SRC; MAL; TLR1; ATP1B1; ABCA1; S1PR1; GRIP1; LRRK2; HPSE; PLSCR1; AKAP6; CD36; PSEN2; TLR2; HCK; ATP1B3; CTNNA1; CD14; LRP6 |
| GO:0005774 | vacuolar membrane | 26/679 | 0.051878459 | CTSA; GNB4; SLC2A6; ATP6V1B2; WDFY3; FPR1; CD1D; RILP; TLR8; NFAM1; TMEM150B; NEU1; MGST1; MCOLN1; HPSE; DRAM1; SORT1; VMP1; MYO7A; TLR7; IFITM3; ANPEP; PSEN2; CD63; RRAGD; LAMP3 |
| MF |  |  |  |  |
| GO:0003779 | actin binding | 32/671 | 0.021300089 | CDK5R1; HOOK1; TRPV4; TPM4; MARCKS; EPB41L3; GCSAM; KLHL3; PLEKHH2; WASF1; SPIRE1; MEFV; GAS2L3; ABLIM1; LRRK2; T2DMTN; PFN2; PSTPIP2; MYO7A; SYNPO; FMNL2; TTN; AIF1; GBP1; SNCA; GAS7; MAP1A; CTNNA1; GAS2L1; PRKN; MYO10; TNNI2 |
| GO:0042277 | peptide binding | 24/671 | 0.033799923 | FZD5; C1QA; CD209; TOMM40L; LILRB2; APBB3; HSPG2; CD1D; MSR1; APBB1; MARCO; PPARG; TMEM158; CLU; FPR2; MGST1; TLR1; LDLRAD3; TLR4; ANPEP; CD36; PGRMC1; TLR2; CD14 |
| GO:0001540 | amyloid-beta binding | 15/671 | 4.03373E-05 | FZD5; C1QA; LILRB2; APBB3; HSPG2; MSR1; APBB1; MARCO; CLU; FPR2; LDLRAD3; TLR4; CD36; PGRMC1; TLR2 |
| GO:0140375 | immune receptor activity | 15/671 | 0.01985406 | IFNGR2; IL31RA; LILRB2; FPR1; CCR7; CCR1; FPR2; IL17RC; FCER1G; CR2; LILRB1; IL15RA; CCRL2; FCGR1B; CSF2RB |
| GO:0038024 | cargo receptor activity | 12/671 | 0.003228245 | LRP12; ASGR2; ASGR1; MSR1; MARCO; FPR2; ABCA1; STAB1; CD36; CD163; SCARF1; LRP6 |
| GO:0008013 | beta-catenin binding | 11/671 | 0.027235747 | PTPRK; TCF7; LEF1; AXIN2; DACT1; TCF7L2; CDH26; GRIP1; KANK1; CTNNA1; PRKN |
| GO:0038187 | pattern recognition receptor activity | 9/671 | 4.03373E-05 | FCN1; TLR8; MARCO; CLEC4E; TLR7; TLR4; CD36; TLR2; CD14 |
| GO:0005080 | protein kinase C binding | 8/671 | 0.049374253 | PKP2; TRPV4; MARCKS; ADAM9; DACT1; SRC; NELL2; PLEK |
| GO:0035325 | Toll-like receptor binding | 5/671 | 0.008088987 | MYD88; TLR1; CD36; S100A9; TLR2 |
| GO:0031720 | haptoglobin binding | 4/671 | 0.027235747 | HBA2; HBB; HBD; HBA1 |
