# Supplementary material for: Integrated multi-omics profiling reveals immune-related biomarkers and regulatory networks for early prediction of tuberculosis in type 2 diabetes mellitus
Source: Front Immunol. 2026 Feb 26;17:1755184. doi: 10.3389/fimmu.2026.1755184 (PMC12979386; doi:10.3389/fimmu.2026.1755184)
Supplement: Supplementary file 5 [file Table4.docx]

Table S4. KEGG enrichment of differential mRNAs in T2DM-TB vs T2DM groups.

| ID | Term | Count | *P*-value | Genes |
| --- | --- | --- | --- | --- |
| hsa05152 | Tuberculosis | 21/386 | 3.54438E-05 | IFNGR2; STAT1; FCGR2A; CD209; RIPK2; CTSS; MYD88; CLEC4E; SRC; FCER1G; IL23A; TLR1; ITGAX; IL10; VDR; TLR4; FCGR1A; CARD9; TLR2; SPHK1; CD14 |
| hsa04148 | Efferocytosis | 20/386 | 1.49644E-05 | TIMD4; C1QA; ADAM9; PPARG; GAS6; ATP8A2; ABCA1; S1PR1; HIF1A; HAVCR1; DOCK1; IL10; STAB1; CD36; MERTK; MFGE8; SCARF1; SPHK1; SIRPA; C1QB |
| hsa04380 | Osteoclast differentiation | 18/386 | 4.27015E-05 | IFNGR2; STAT1; OSCAR; FCGR2A; LILRA5; LILRB2; NCF2; PPARG; LILRA2; LILRB3; CAMK4; LILRB1; LILRA1; SPI1; FCGR1A; GAB2; LILRB4; SIRPA |
| hsa04820 | Cytoskeleton in muscle cells | 18/386 | 0.012741492 | VCAN; PKP2; LMNB1; COL6A3; MYL9; HSPG2; TPM4; SDC3; SDC2; ATP1B1; TNNT1; FMNL2; TTN; DSC2; OBSCN; ITGA2B; ATP1B3; TNNI2 |
| hsa04621 | NOD-like receptor signaling pathway | 17/386 | 0.003656862 | STAT1; NAMPT; RIPK2; CTSB; BCL2L1; GBP4; MYD88; MEFV; NAIP; GBP5; GBP1; IRF7; TLR4; CARD9; GBP2; CASP5; CYBB |
| hsa05417 | Lipid and atherosclerosis | 17/386 | 0.013382292 | ICAM1; SOD2; VCAM1; NCF2; BCL2L1; PPARG; MYD88; SRC; ABCA1; IRF7; TLR4; TNFSF10; CD36; TLR2; CYBB; HSPA6; CD14 |
| hsa04142 | Lysosome | 16/386 | 0.000192527 | CTSA; NPC2; CTSL; ASAH1; SLC11A1; CTSS; CTSF; CTSB; NEU1; GM2A; MCOLN1; SORT1; GNS; T2DMXL2; CD63; LAMP3 |
| hsa04145 | Phagosome | 15/386 | 0.003506722 | CTSL; FCGR2A; CD209; ATP6V1B2; CTSS; MSR1; NCF2; RILP; MARCO; TLR4; FCGR1A; CD36; TLR2; CYBB; CD14 |
| hsa05225 | Hepatocellular carcinoma | 15/386 | 0.00731823 | FZD5; TCF7; TXNRD3; LEF1; AXIN2; E2F2; BCL2L1; E2F1; TCF7L2; MGST1; SMARCD3; WNT7A; FZD1; GSTO1; LRP6 |
| hsa05167 | Kaposi sarcoma-associated herpesvirus infection | 15/386 | 0.024362318 | GNB4; GNG10; STAT1; ICAM1; TCF7; LEF1; E2F2; CD86; CCR1; E2F1; TCF7L2; SRC; HIF1A; IRF7; HCK |
| hsa04625 | C-type lectin receptor signaling pathway | 12/386 | 0.001997844 | STAT1; IRF1; EGR2; CD209; CLEC4E; SRC; FCER1G; IL23A; IL10; EGR3; CARD9; BCL3 |
| hsa05418 | Fluid shear stress and atherosclerosis | 12/386 | 0.020158134 | CTSL; ICAM1; VCAM1; MAPK7; TRPV4; NCF2; SDC2; SRC; MGST1; ACVR2B; ITGA2B; GSTO1 |
| hsa05144 | Malaria | 11/386 | 7.60812E-06 | HBA2; ICAM1; HBB; VCAM1; MYD88; SDC2; IL10; TLR4; CD36; HBA1; TLR2 |
| hsa04620 | Toll-like receptor signaling pathway | 11/386 | 0.00782694 | STAT1; TLR8; CD86; MYD88; TLR1; TLR7; CXCL10; IRF7; TLR4; TLR2; CD14 |
| hsa04936 | Alcoholic liver disease | 11/386 | 0.049949446 | C1QA; C2; TCF7; LEF1; MYD88; TCF7L2; IL17RC; ADIPOR1; TLR4; CD14; C1QB |
| hsa05140 | Leishmaniasis | 10/386 | 0.001789187 | IFNGR2; STAT1; FCGR2A; NCF2; MYD88; IL10; TLR4; FCGR1A; TLR2; CYBB |
| hsa05133 | Pertussis | 10/386 | 0.001974563 | IRF1; C1QA; C2; SERPING1; MYD88; IL23A; IL10; TLR4; CD14; C1QB |
| hsa04610 | Complement and coagulation cascades | 10/386 | 0.004818414 | F13A1; C1QA; C2; SERPING1; CLU; SERPINB2; ITGAX; CR2; SERPINA1; C1QB |
| hsa04662 | B cell receptor signaling pathway | 10/386 | 0.006114071 | PIK3AP1; BANK1; LILRA5; LILRB2; LILRA2; LILRB3; CR2; LILRB1; LILRA1; LILRB4 |
| hsa04666 | Fc gamma R-mediated phagocytosis | 10/386 | 0.010189096 | FCGR2A; MARCKS; WASF1; LIMK2; DOCK1; FCGR1A; GAB2; HCK; SPHK1; MYO10 |
| hsa04064 | NF-kappa B signaling pathway | 10/386 | 0.016064452 | ICAM1; TNFSF13B; VCAM1; EDAR; BCL2L1; MYD88; BCL2A1; TLR4; CD14; LTBR |
| hsa05143 | African trypanosomiasis | 9/386 | 2.18972E-05 | HBA2; ICAM1; HBB; VCAM1; MYD88; APOL1; IL10; F2RL1; HBA1 |
| hsa05221 | Acute myeloid leukemia | 9/386 | 0.002627451 | TCF7; LEF1; PIM1; TCF7L2; BCL2A1; DUSP6; SPI1; FCGR1A; CD14 |
| hsa05323 | Rheumatoid arthritis | 9/386 | 0.021125934 | CTSL; ICAM1; TNFSF13B; ATP6V1B2; IL15; CD86; IL23A; TLR4; TLR2 |
| hsa05150 | Staphylococcus aureus infection | 9/386 | 0.030146911 | FCGR2A; ICAM1; C1QA; C2; FPR1; FPR2; IL10; FCGR1A; C1QB |
| hsa04066 | HIF-1 signaling pathway | 9/386 | 0.048242578 | IFNGR2; PFKFB3; HK3; HIF1A; TIMP1; HK2; TLR4; CYBB; LTBR |
| hsa05412 | Arrhythmogenic right ventricular cardiomyopathy | 8/386 | 0.033530728 | PKP2; TCF7; LEF1; TCF7L2; SLC8A1; DSC2; ITGA2B; CTNNA1 |
| hsa04520 | Adherens junction | 8/386 | 0.049678329 | MYL9; TCF7; LEF1; WASF1; TCF7L2; SRC; NECTIN2; CTNNA1 |
| hsa00480 | Glutathione metabolism | 7/386 | 0.012593897 | LAP3; OPLAH; GPX1; MGST1; PGD; ANPEP; GSTO1; |
| hsa04978 | Mineral absorption | 7/386 | 0.016356988 | SLC31A1; ATP1B1; VDR; FTH1; SLC8A1; MT2A; ATP1B3 |
| hsa05217 | Basal cell carcinoma | 7/386 | 0.019269678 | FZD5; TCF7; LEF1; AXIN2; TCF7L2; WNT7A; FZD1 |
| hsa05219 | Bladder cancer | 6/386 | 0.008177834 | E2F2; E2F1; TYMP; SRC; DAPK1; HBEGF |
| hsa04672 | Intestinal immune network for IgA production | 6/386 | 0.019018241 | TNFSF13B; IL15; CD86; IL10; IL15RA; LTBR |
| hsa05134 | Legionellosis | 6/386 | 0.034309454 | MYD88; NAIP; TLR4; TLR2; HSPA6; CD14 |
| hsa00532 | Glycosaminoglycan biosynthesis - chondroitin sulfate / dermatan sulfate | 4/386 | 0.011839841 | CSGALNACT1; CHST15; DSE; DSEL |
